# Switch of serotonergic descending inhibition into facilitation by a spinal chloride imbalance in neuropathic pain

**DOI:** 10.1126/sciadv.abo0689

**Published:** 2022-07-27

**Authors:** Franck Aby, Louis-Etienne Lorenzo, Zoé Grivet, Rabia Bouali-Benazzouz, Hugo Martin, Stéphane Valerio, Sara Whitestone, Dominique Isabel, Walid Idi, Otmane Bouchatta, Philippe De Deurwaerdere, Antoine G. Godin, Cyril Herry, Xavier Fioramonti, Marc Landry, Yves De Koninck, Pascal Fossat

**Affiliations:** ^1^ Université de Bordeaux, Bordeaux, France.; ^2^ Institut des maladies neurodégénératives (IMN), CNRS UMR 5293, Bordeaux, France.; ^3^ CERVO Brain Research Center, Université Laval, Québec City, Canada.; ^4^ Department of Psychiatry and Neuroscience, Université Laval, Québec City, Canada.; ^5^ NutriNeuro, UMR, INRAe, 1286 Bordeaux, France.; ^6^ Aquineuro, SA, Bordeaux, France.; ^7^ Université Cadi Ayyad, Marrakech, Morocco.; ^8^ Institut des neurosciences cognitives et intégratives d’aquitaine (INCIA) CNRS UMR 5287, Bordeaux, France.; ^9^ Neurocentre Magendie, INSERM, U862, Bordeaux, France.

## Abstract

Descending control from the brain to the spinal cord shapes our pain experience, ranging from powerful analgesia to extreme sensitivity. Increasing evidence from both preclinical and clinical studies points to an imbalance toward descending facilitation as a substrate of pathological pain, but the underlying mechanisms remain unknown. We used an optogenetic approach to manipulate serotonin (5-HT) neurons of the nucleus raphe magnus that project to the dorsal horn of the spinal cord. We found that 5-HT neurons exert an analgesic action in naïve mice that becomes proalgesic in an experimental model of neuropathic pain. We show that spinal KCC2 hypofunction turns this descending inhibitory control into paradoxical facilitation; KCC2 enhancers restored 5-HT–mediated descending inhibition and analgesia. Last, combining selective serotonin reuptake inhibitors (SSRIs) with a KCC2 enhancer yields effective analgesia against nerve injury–induced pain hypersensitivity. This uncovers a previously unidentified therapeutic path for SSRIs against neuropathic pain.

## Teaser

Switch of serotonin descending inhibition into excitation in neuropathic pain reveals a treatment combining SSRI/KCC2 enhancers.

## INTRODUCTION

Pain is a warning system that protects organisms against real or potential tissue damages. This unpleasant feeling can be efficiently reduced in case of excessive pain thanks to the existence of an endogenous system that controls pain transmission. Yet, the brain’s ability to control pain sensitivity is equally important to allow fight or flight responses. There is growing evidence that a failure of endogenous analgesia may underlie some pathological pain hypersensitivity syndromes (*1*). Endogenous pain control involves descending pathways that directly or indirectly project to the dorsal horn of the spinal cord (DHSC) (*2*–*5*). Indirect projections involve different brainstem nuclei such as the periaqueductal gray (PAG) and the rostral ventromedial medulla (RVM) (*2*, *6*). These regions also receive the ascending nociceptive information coming from the DHSC and exert a potent descending inhibition. This ascending-descending loop is highlighted in the phenomenon of diffuse nociceptive inhibitory control, more recently referred to as conditioned pain modulation (CPM) in human (*2*, *7*). A deficit in CPM has emerged as a good predictor of poor pain outcome after surgery and growing lines of evidence link abnormal CPM to chronic pain states in patients with postoperative, neuropathic, or idiopathic pain. Therefore, CPM paradigms are commonly used in clinics to evaluate pain status (*8*–*11*). Alterations in CPM reflect an imbalance between facilitatory and inhibitory descending pain pathways to the DHSC, but the underlying neural circuits remain largely unknown (*3*, *12*). Recent studies have identified specific neural circuits in the PAG and the RVM promoting either pain inhibition or pain facilitation (*13*–*17*). These circuits involve monoaminergic neurons that are also the target of the more efficient current medication against chronic pain, namely, the serotonin-noradrenaline reuptake inhibitors (SNRIs). In contrast, selective serotonin reuptake inhibitors (SSRIs) have failed to show analgesic efficacy in neuropathic pain (*18*–*20*). Yet, serotonin (5-HT) neurons of the brainstem represent an important projection to the DH and are known as pain modulators (*21*–*24*). 5-HT–mediated control of nociceptive transmission varies between pathophysiological conditions, suggesting the presence of both 5-HT descending facilitation and inhibition in variable balance (*3*, *15*, *16*, *25*).

In the present study, we aimed to uncover the mechanism underlying the lability of endogenous serotoninergic pain modulation by selective optogenetic manipulation of 5-HT descending pathways in naïve and neuropathic animals. We targeted 5-HT neurons of the RMg that are known to project to the DHSC. We found that this specific population mediates a descending inhibition through activation of local spinal inhibitory interneurons. This inhibitory circuit is turned into facilitation due to a spinal chloride dysregulation in animals with nerve injury. We show that normalizing chloride homeostasis allows for the recovery of 5-HT descending inhibition and confers analgesic efficacy of SSRIs against neuropathic pain.

## RESULTS

### Optogenetic activation of RMg 5-HT neurons causes selective spinal 5-HT release

**Fig. 1.**
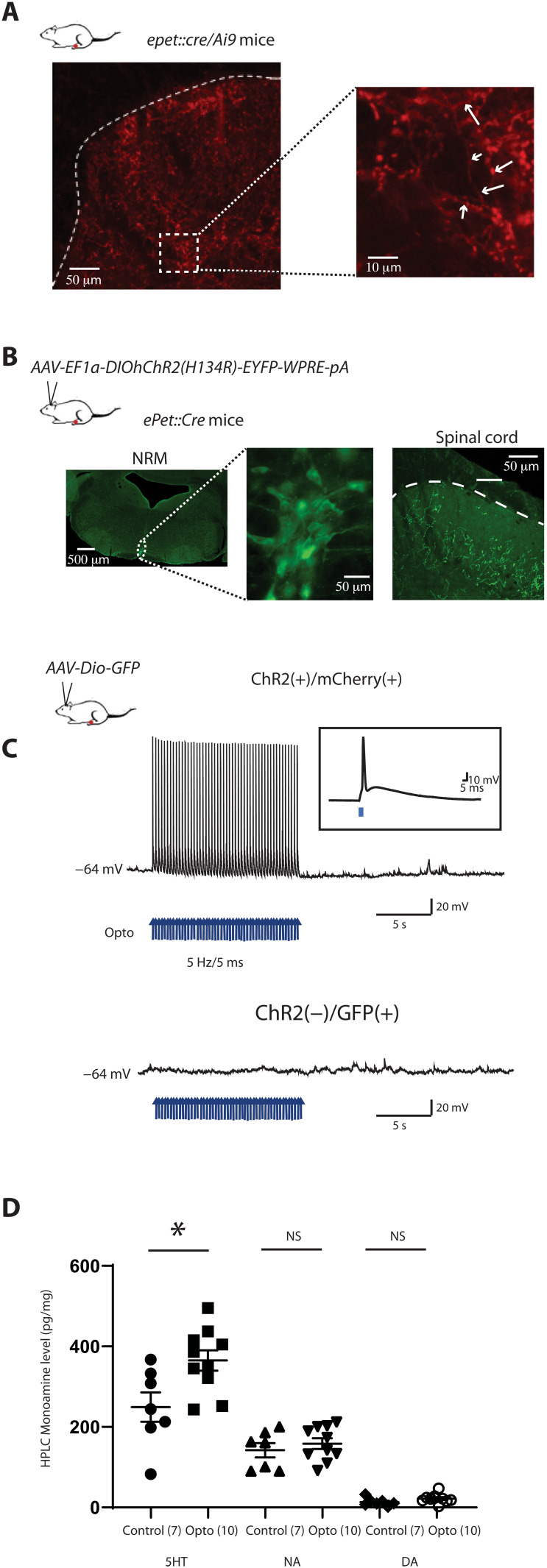
Optogenetic manipulation of 5-HT RMg neurons induces 5-HT release on DHSC. (**A**) Crossbreeding of *ePet::Cre* mice with an *Ai9::tdtomato* reporter mice allows the localization of cre-recombinase expression in 5-HT fibers. Dense tomato staining is observed in the superficial and deep lamina of the DHSC. Inset: arrows show clear immunopositive fibers with putative axonic buttons. (**B**) Stereotaxic injection of *AAV-EF1a-DIOhChR2(H134R)-EYFP-WPRE-pA* in RMg of *ePet::Cre* mice induces GFP expression in 5-HT neurons and 5-HT fibers in the deep lamina of the DHSC. (**C**) Patch-clamp recordings of ChR2(+)/GFP(+) neurons in RMg slices of infected *ePet::Cre* mice. Blue light flash at 5 Hz/5 ms induced depolarization and action potential firing of 5-HT neurons. The same stimulation in ChR2(−)/GFP(+) neurons has no effect. (**D**) HPLC measurement from DHSC after 5 Hz/5 ms optogenetic stimulation of 5-HT fibers induced a significant increase in 5-HT levels but not in other monoamines, i.e., dopamine and noradrenaline. HPLC, High-Performance Liquid Chromatography.

Our first aim was to clearly establish the role of acute stimulation of 5-HT neurons of the RMg in nociceptive transmission. We used a specific mouse strain expressing cre-recombinase under the control of a PET1 (polyethylene terephthalate *pheochromocytoma 12 ETS (E26 transformation-specific*) promoter specific to 5-HT neurons (*26*). We first crossbred *ePet::Cre* mice with Ai9::tdtomato reporter mice. We observed a high level of colocalization of cre-recombinase with tryptophan hydroxylase 2 (TPH2), a specific marker of 5-HT neurons (fig. S1). We also observed spinal projections of cre(+) fibers in the dorsal horn in two main regions, the superficial and deep laminae (Fig. 1A). Higher magnification showed clear putative synaptic bouton in the deep layer of the DHSC (Fig. 1A, inset). Next, we used a viral-based transduction approach to express the excitatory opsin channelrhodopsin 2 (ChR2) in 5-HT neurons. Three weeks after injection of an adeno-associated virus (AAV) containing ChR2 and enhanced yellow fluorescent protein (EYFP) [*AAV-EF1a-DIOhChR2(H134R)-EYFP-WPRE-pA*] in the RMg, we found EYFP expression in both cell bodies of 5-HT neurons of the RMg and their projections to the DHSC (fig. S1B and Fig. 1B). We used slices of RMg to perform whole-cell patch clamp recordings to determine the validity of opsin expression and light stimulation protocol (Fig. 1C). We used a stimulation protocol that activates 5-HT neurons in a physiological range (5 Hz/5 ms) (*27*), and we observed that neurons fired action potentials that faithfully followed optogenetic stimulation (Fig. 1C, inset). In the next step, we used high-performance liquid chromatography (HPLC) measurements of the DHSC after optogenetic stimulation of 5-HT neurons, and we observed that our protocol elicited a significant increase in the level of 5-HT in the DHSC, but not of the other monoamines measured (Fig. 1D).

### Optogenetic activation of spinal RMg 5-HT fibers causes analgesia

**Fig. 2.**
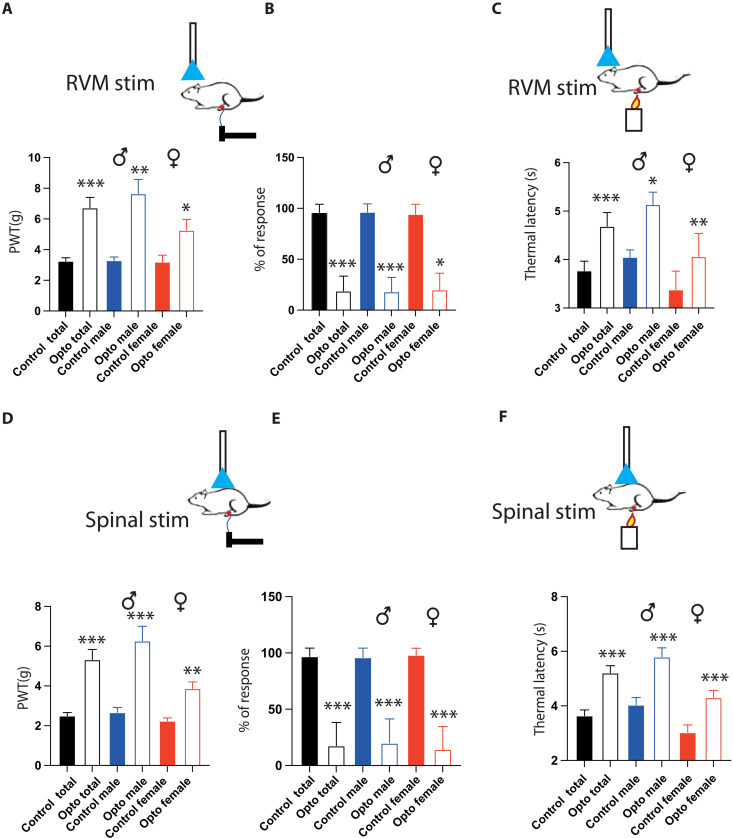
Optogenetic stimulation of 5-HT neurons induces a sex-independent mechanical and thermal analgesia. (**A**) PWT is significantly increased (total: 3.3 ± 0.21 g before and 6.7 ± 0.68 g during opto, *N* = 18; males: 3.3 ± 0.23 before and 7.7 ± 0.93 g during opto, *N* = 11; females: 3.2 ± 0.44 g before and 5.3 ± 0.7 g, *N* = 7). (**B**) RS is significantly decreased (total: 95.6 ± 2% before and 18.9 ± 3.4% during opto, *N* = 18; males: 96.36 ± 2.44% before and 18.2 ± 4% during opto, *N* = 11; females: 94.3 ± 3.7% before and 20 ± 6.1%, *N* = 7). (**C**) TL is significantly decreased (total: 3.8 ± 0.2 s before and 4 ± 0.3 s during opto, *N* = 15; males: 4 ± 0.15 s before and 5.1 ± 0.26 s during opto, *N* = 7; females: 3.4 ± 0.389 s before and 4 ± 0.48 s, *N* = 8). (**D**) Optogenetic stimulation of 5-HT fibers induces a sex-independent mechanical and thermal analgesia. (**E**) PWT is significantly increased (total: 2.5 ± 0.16 g before and 5.3 ± 0.5 g during opto, *N* = 28; males: 2.7 ± 0.24 before and 6.3 ± 0.73 g during opto, *N* = 17; females: 2.2 ± 0.15 g before and 3.9 ± 0.33 g, *N* = 11). (**F**) RS is significantly decreased (total: 96.9 ± 1.4% g before and 17.7 ± 4% during opto, *N* = 26; males: 96 ± 1.8% before and 20 ± 5.5% during opto, *N* = 15; females: 98.2 ± 1.8% before and 14.6 ± 6%, *N* = 11). (D) TL is significantly decreased (total: 3.6 ± 0.21 g before and 5.2 ± 0.26 g during opto, *N* = 28; males: 4 ± 0.26 before and 5.8 ± 0.33 g during opto, *N* = 17; females: 3 ± 0.27 g before and 4.3 ± 0.26 g, *N* = 11). *P < 0.05, **P < 0.01, and ***P < 0.001, Wilcoxon matched-pair signed rank test.

Then, we performed optogenetic stimulation of 5-HT neurons in both male and female animals using our stimulation protocol. ChR2 activation with optic fibers implanted above the injection site (i.e., RMg) (see Materials and Methods) elicited a strong mechanical and thermal analgesia (Fig. 2, A and B). We show that (i) paw withdrawal threshold (PWT) was significantly increased (fig. S2A1), (ii) five repetitive stimulations (RSs) with a suprathreshold Von Frey filament failed to induce reproducible behavioral responses (fig. S2A2), and (iii) thermal latency (TL) to paw withdrawal was significantly increased (Fig. 2B). These effects were equivalent in males and females. To focus on descending pathways projecting to the DHSC, we performed the same experiments but implanted the optic fiber above the lumbar spinal cord to specifically target 5-HT projecting fibers (*28*). As shown in Fig. 2 (C and D), we observed the same results as in Fig. 1 (A and B), suggesting that 5-HT–induced analgesia is due to 5-HT fibers projecting in DHSC. As a control, optogenetic stimulation in ChR2(−) mice remained ineffective regardless of the location of the fiber implantation (fig. S2).

### RMg 5-HT neurons inhibit spinal nociception

**Fig. 3.**
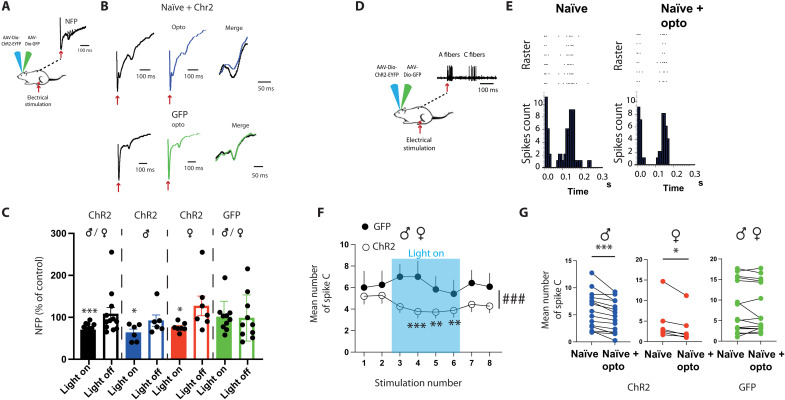
Optogenetic activation of 5-HT descending fibers alters dorsal horn neuron responses to peripheral stimulations. (**A**) Field potential recordings in mice. (**B**) NFPs are decreased during optogenetic stimulation of 5-HT neurons in Chr2(+) but not modified in ChR2(−) mice [ChR2(+): 70 ± 5% of control during opto, *N* = 12. ChR2(−): 101 ± 12% of control during opto, *N* = 10]. (**C**) Decrease in NFP is similar in males and females (males: 63.7 ± 8.2% of control during opto, *N* = 6; females: 76.5 ± 4% of control during opto, *N* = 7). *P < 0.05 and ***P < 0.001, one-sample *t* and Wilcoxon test. (**D**) Single-unit recordings in the DHSC. (**E**) Poststimulus histogram shows that optogenetic stimulation of 5-HT neurons in ChR2(+) mice induced a decrease in C-fiber component. (**F**) Time course of optogenetic stimulation showing a significant decrease in the number of C-spike induced by electric stimulation of the peripheral receptive field in ChR2(+) but not in ChR2(−) mice. RM ANOVA, *P* < 0.001 and *F* = 8.008, *N* = 25, ** and ***, HolmSidak post hoc compared to stimulation 1 and 2 before opto. ###, Two-way ANOVA: GFP [ChR2(−) compared to ChR2(+), *P* < 0.001]. (**G**) During optogenetic stimulation, C-fiber response is decreased in a similar manner in males and females (males: 5.9 ± 0.9 spikes before and 4.7 ± 0.74 spikes during opto, *N* = 15; females: 4.8 ± 2 spikes before and 3.5 ± 1.6 spikes during opto, *N* = 6). In ChR2(−) mice, optogenetic stimulation has no effect (8.3 ± 1.6 spikes before and 8 ± 1.5 spikes during opto, *N* = 15). *P < 0.05 and ***P < 0.001, Wilcoxon matched-pair signed rank test.

To confirm that such analgesia is due to direct modification of nociceptive transmission in DHSC, we performed in vivo electrophysiological recordings. We first evaluated the global consequence of optogenetic stimulation of 5-HT neurons by recording local field potential after peripheral electrical stimulation of the paw (Fig. 3, A to C). Nociceptive field potentials (NFPs) were decreased during optogenetic stimulation in mice expressing ChR2, but not in mice expressing green fluorescent protein (GFP) only. This effect was equivalent in males and females and confirmed that 5-HT–induced analgesia occurs through a modification of the nociceptive transmission in the DHSC. To determine whether the decrease in nociceptive information is localized at the level of the DHSC, we recorded deep dorsal horn neurons that project outside DHSC. We restricted recordings to wide dynamic range neurons (WDRs) that are highly convergent and exhibit a clear C-component, i.e., a delayed response following suprathreshold electric stimulation of the corresponding receptive field, which can be used as a readout of nociceptive transmission (*29*) (Fig. 3, D to G). During optogenetic stimulation, we observed a decrease in the number of action potentials in the C-fiber range (Fig. 3, E and F) that was not present with mice expressing the GFP tag only. This decrease was equivalent in males and females (Fig. 3G).

### RMg 5-HT analgesia works through local spinal GABAergic/glycinergic neurons

**Fig. 4.**
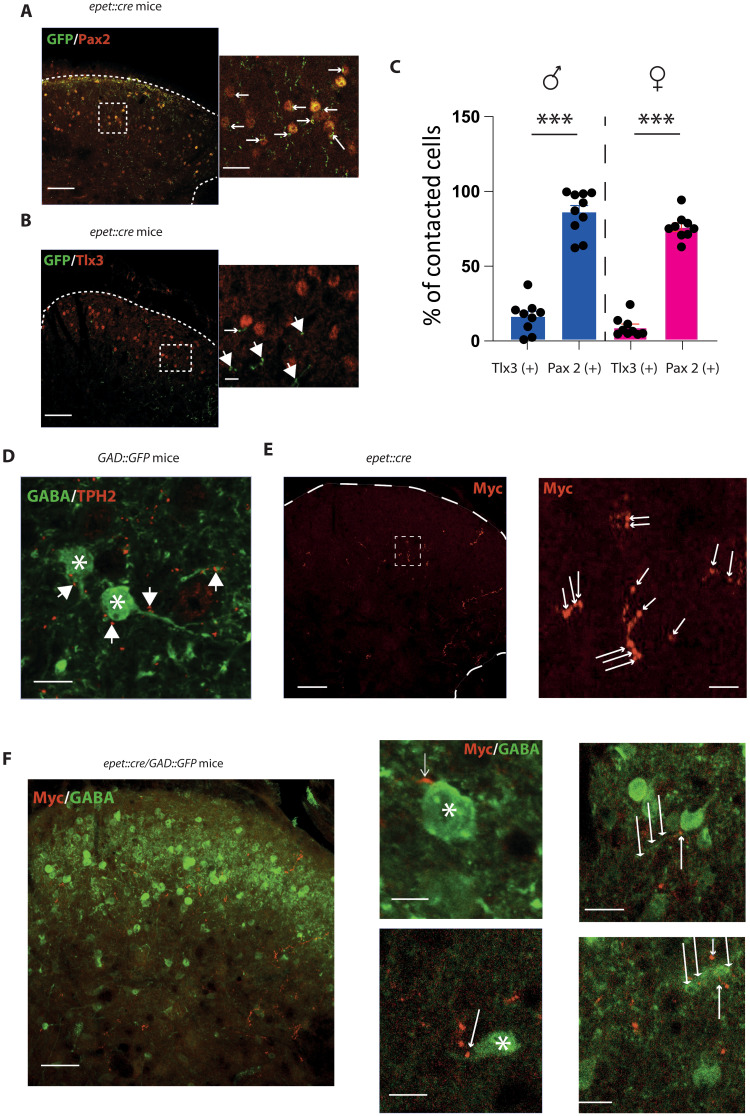
5-HT descending fibers project to spinal inhibitory interneurons. (**A**) GFP (green) and PAX2 (red) immunolabeling in the DH. Scale bar, 100 μm. Inset: high magnification showing contacts between Pax2 and 5-HT fibers. Scale bar, 50 μm. (**B**) GFP (green) and Tlx3 (red) immunolabeling in the DH. Scale bar, 100 μm. Inset: high magnification, low or no contact. Scale bar, 25 μm. (**C**) After 3D reconstruction (see Materials and Methods) quantification of contacts between cell bodies (Pax2 and Tlx3) and 5-HT fibers. (**C**) Contacted cells are significantly higher for Pax2(+) than for Tlx3(+) in both males and females [males (blue): 86.2 ± 4.6% for Pax2 and 15.5 ± 3.8% for Tlx3, *P* < 0.001, Mann-Whitney; females (red): 76.3 ± 2.9% for Pax2 and 8.3 ± 2.2% for Tlx3, *P* < 0.001, Mann-Whitney]. (**D**) Immunostaining against TPH2 in slices of lumbar spinal cord of *GAD67::GFP* mice. Scale bar, 25 μm. GABA neurons [GFP(+) in green, stars] or fibers in DH are contacted by TPH2-positive putative synaptic button (in red, white arrows). (**E**) Expression of Myc-tag in synaptic 5-HT terminals in DH following injections of an AAV-synaptophysin-Myc. Immunoreactivity is present in the deeper lamina of the DHSC. Scale bar, 100 μm. Higher magnification shows 5-HT axonic button (white arrow). Scale bar, 20 μm. (**F**) Myc(+) synaptic buttons are in direct contact with GAD67-positive cells and fibers in *epet::cre/ GAD67::GFP* mice. Scale bar, 20 μm. Inset (top): Myc(+) button on GAD(+) cell bodies. Inset (bottom): Myc(+) button on GAD(+) on GAD(+) fibers.

**Fig. 5.**
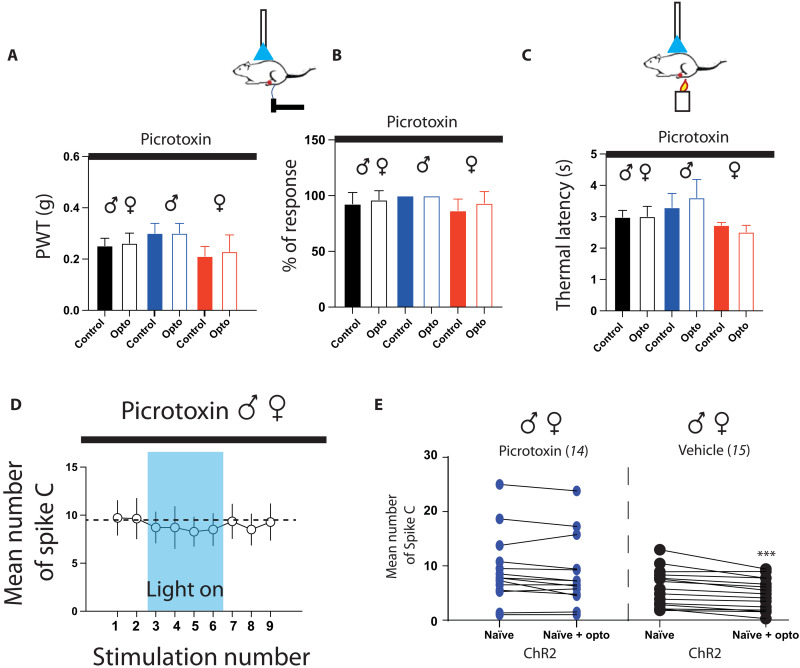
Blocking GABA_A_ receptors prevents the analgesic role of descending 5-HT fibers. Picrotoxin suppresses mechanical analgesia induced by optogenetic stimulation of 5-HT neurons. (**A**) PWT is not modified by optogenetic stimulation in both males and females (*P* > 0.05 for total, male, and female). (**B**) RS is not altered (*P* > 0.05 for total, male, and female). (**C**) Picrotoxin suppresses the thermal analgesia induced by optogenetic stimulation of the 5-HT neurons. TL remained unmodified in both males and females (*P* > 0.05 for total, male, and female, *N* = 11, 5, and 6, respectively). Wilcoxon matched-pair signed rank test. (**D**) Picrotoxin suppresses the decrease in WDR neuron response to C-fibers induced by optogenetic stimulation of 5-HT neurons (*P* > 0.05, RM ANOVA). (**E**) Mean number of spike C is not modified during optogenetic stimulation of 5-HT neurons under picrotoxin, although the same stimulation decreases C-fiber response with vehicle (picrotoxin; 9.1 ± 1.7 spikes before and 8.6 ± 1.7 spikes during opto, *N* = 14; vehicle; 5.8 ± 0.9 spikes before and 4.7 ± 0.74 spikes during opto, *N* = 15). ***P < 0.001, Wilcoxon matched-pair signed rank test.

Subsequently, we worked to determine the spinal microcircuit involved in this analgesia. 5-HT receptors are widely expressed in the DHSC and exert excitatory or inhibitory influences on either excitatory or inhibitory spinal neurons. Although it is known that 5-HT can activate inhibitory neurons (*30*, *31*), we cannot exclude that analgesia would be due to a direct inhibitory action of 5-HT fibers on excitatory afferent fibers or projection neurons. We first aimed at determining if 5-HT neurons could also express inhibitory neurotransmitters. Most of the inhibitory interneurons in the RVM including RMg express both glycine and GABA (*32*), and we used *GAD67::GFP* mice to determine whether GFP fluorescence could be colocalized with TPH2 staining in the RMg and found no colocalization (fig. S3A). We also used *ePet::Cre*-Ai9::tdtomato mice (see above) and GAD65 immunostaining and found numerous putative synaptic contacts of GABA terminals on 5-HT neurons and fibers in the RMg but no colocalization between GABA and 5-HT fibers/terminals neither in the RMg nor in the DHSC (fig. S3, B and C). Then, we sought for 5-HT targets in the DHSC and performed immunostaining of both excitatory and inhibitory interneurons in spinal slices of *epet::cre* mice injected in the RMg with an AAV-CAG-Flex-GFP. We compared the apposition of GFP fluorescence in 5-HT fibers with a marker of excitatory (tlx3) or inhibitory neurons (Pax2) (Fig. 4, A to C). We found that 5-HT fibers are in closer contact with Pax2 than Tlx3 in both males and females (Fig. 4C), suggesting connections between 5-HT fibers and local inhibitory interneurons. To confirm this hypothesis, we used *GAD67::GFP* mice and performed TPH2 immunostaining. We found putative 5-HT(+) boutons onto GABAergic interneurons on both cell bodies and fibers (Fig. 4D). We injected an AAV-DIO-Synaptophysin-myc in the RMg of *ePET::cre* and found synaptic terminals in the deep lamina of the DHSC (Fig. 4E). Last, we crossbred the *ePET::cre* mouse with the *GAD67::GFP* mouse to seek for synaptic buttons between 5-HT fibers originating in the RMg and GABAergic neurons in the DHSC. We injected an AAV-DIO-Synaptophysin-myc in the RMg and performed a co-immunohistochemistry of Myc tag and GFP. We observed synaptic contacts between 5-HT fibers and GABAergic neurons on both cell bodies and fibers (Fig. 4F). To determine whether our optogenetic protocol activates inhibitory interneurons in the DHSC, we performed optogenetic stimulation of 5-HT descending fibers in anesthetized mice. One hour after optogenetic stimulation, mice were euthanized and quickly perfused, and early gene cFos immunostaining was performed (fig. S4). To identify inhibitory interneurons, an immunostaining against Pax2 was also conducted. We found, in both superficial and deep layer of the DHSC, c-Fos (+) neurons that were also Pax2(+), showing that optogenetic activation of 5HT fibers activate spinal inhibitory interneurons. Last, to confirm that the analgesic effect was due to the microcircuit linking 5-HT neurons of the RMg and inhibitory interneurons in the DHSC, we evaluated mechanical and thermal pain sensitivity during optogenetic stimulation of 5-HT fibers after intrathecal injection of picrotoxin to block GABA_A_ and glycine (Gly) receptors. Picrotoxin suppressed the analgesic effect of optogenetic stimulation in both males and females (Fig. 5, A and B). To confirm that this blockade altered nociceptive transmission in DHSC, we performed in vivo electrophysiological recordings of WDR neurons, and we observed that blockade of GABA/Gly receptors suppressed the decrease in WDR neuron responsiveness (Fig. 5, C and D). Together, these results show that 5-HT fibers come from the RMg synapse onto spinal inhibitory interneurons to, in turn, inhibit spinal nociceptive transmission.

### Spinal KCC2 blockade switches descending RMg 5-HT inhibition into excitation

**Fig. 6.**
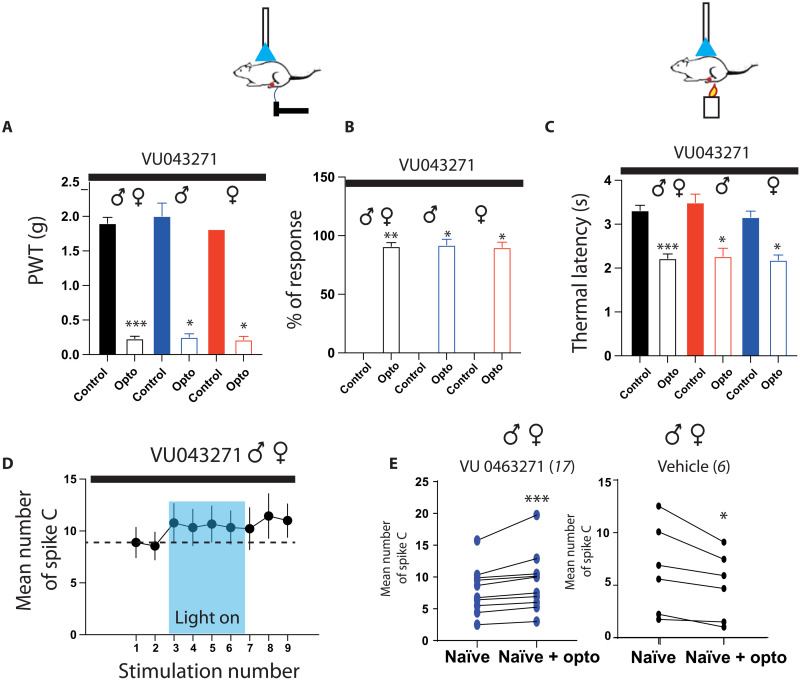
Inhibition of chloride transporters switches 5-HT descending inhibition into facilitation. VU043271 switches the mechanical analgesia induced by optogenetic stimulation of 5-HT neurons into mechanical hyperalgesia and allodynia. (**A**) PWT is significantly decreased by optogenetic stimulation in both males and females [total (black): 1.9 ± 0.1 g before and 0.23 ± 0.03 during opto, *N* = 12; males (blue): 2 ± 0.19 g before and 0.25 ± 0.05 g during opto, *N* = 6; females (red): 1.8 before and 0.22 ± 0.05 g during opto, *N* = 6]. (**B**) RS is significantly increased [total (black): 0 ± 0 before and 90.9 ± 3.15% during opto, *N* = 11; males (blue): 0 ± 0% before and 93.3 ± 4.9% during opto, *N =* 6; females (red): 0 ± 0% before and 90 ± 4.5% during opto, *N* = 6]. (**C**) VU043271 switches the thermal analgesia induced by optogenetic stimulation of the 5-HT neurons into hyperalgesia. TL is significantly decreased in both males and females [total (black): 3.3 ± 0.12 s before opto, 2.2 ± 0.1 s during opto, *N* = 11; males (blue): 3.5 ± 0.2 s before and 2.27 ± 0.18 s, *N* = 5; females (red): 3.2 ± 0.13 s before and 2.2 ± 0.11 s, *N* = 6]. (**D**) VU043271 induces an increase in WDR neuron response to C-fibers induced by optogenetic stimulation of 5-HT neurons (*P* < 0.05, Friedman test; *P < 0.05, Dunn’s multiple comparison test). (**E**) Mean number of spike C is increased during optogenetic stimulation of 5-HT neurons under VU043271, although the same stimulation decreases C-fiber response with vehicle (VU043271: 8 ± 0.8 spikes before, 9.3 ± 1 spikes during opto, *N* = 15; vehicle: 7.5 ± 1.9 spikes before, 5.8 ± 1.3 spikes during opto, *N* = 6). *P < 0.05, **P < 0.01, and ***P < 0.001, Wilcoxon matched-pair signed rank test.

In neuropathic pain models, disinhibition due to chloride dysregulation appears to be a crucial mechanism responsible for neuronal hyperexcitability and pain hypersensitivity (*33*–*35*). We then investigated whether we could reverse the effect of the optogenetic stimulation of 5-HT fibers coming from the RMg by changing chloride balance in the DHSC. We performed intratumoral injections of two different blockers of chloride transporters, the nonselective inhibitor furosemide (fig. S5) and the selective K^+^-Cl^−^-transporter KCC2 inhibitor VU043271 (Fig. 6, A and B). In both cases, optogenetic stimulation of 5-HT fibers induced a significant increase in pain sensitivity in both males and females. PWT was significantly decreased (Fig. 6A1 and fig. S5), subthreshold Von Frey filament became suprathreshold (RS, Fig. 6A2 and fig. S5), and TL was significantly decreased (Fig. 6B and fig. S5). In vivo electrophysiological recordings showed that VU043271 caused a switch in the response of WDR neurons to optogenetic manipulation of 5-HT neurons. Under VU043271 superfusion, but not vehicle, WDR neurons increased their response to peripheral stimulation when RMg 5-HT fibers are optogenetically stimulated (Fig. 6, C and D). These results show that altering chloride balance could switch serotoninergic inhibition into excitation.

### Nerve injury switches RMg 5-HT inhibition to excitation via spinal KCC2 hypofunction

**Fig. 7.**
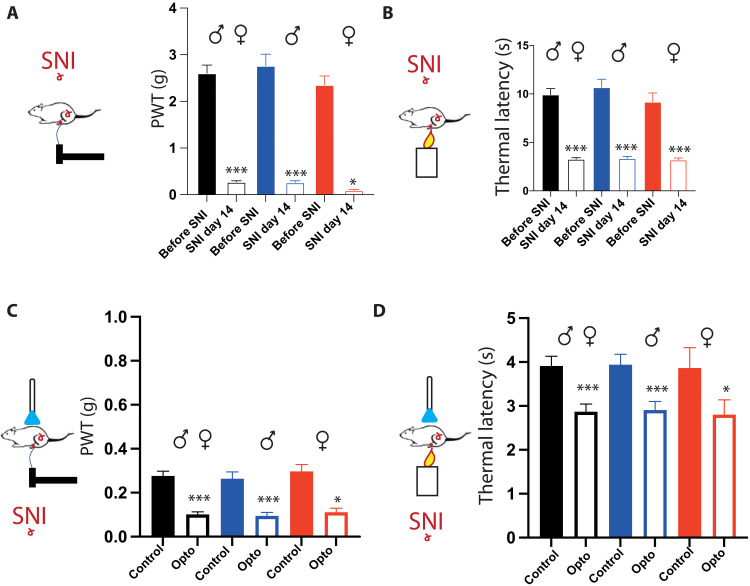
SNI switches 5-HT descending fiber inhibition into facilitation. (**A**) Fourteen days after surgery, the SNI induces a robust mechanical allodynia in both males and females. PWT is significantly reduced in both males and females [total (black): 2.5 ± 0.11 g before and 0.28 ± 0.01 g after surgery; males (blue): 2.7 ± 0.2 g before surgery and 0.28 ± 0.02 g after surgery; females (red): 2.4 ± 0.13 g before surgery and 0.3 ± 0.02 g after surgery]. (**B**) Fourteen days after surgery, the SNI induces a robust thermal hyperalgesia in both males and females. TL is significantly reduced in both males and females [total (black): 9.9 ± 0.63 s before and 3.3 ± 0.14 s after surgery; males (blue): 10.7 ± 0.83 s before surgery and 3.3 ± 0.2 s after surgery; females (red): 9.2 ± 0.92 s before surgery and 3.2 ± 0.2 s after surgery]. (**C**) In SNI mice, optogenetic stimulation of 5-HT neurons induced a significant decrease in PWT [total (black): 0.28 ± 0.02 g before and 0.1 ± 0.02 g, *N* = 18; males (blue): 0.26 ± 0.03 before and 0.09 ± 0.02 g during opto, *N* = 11; females (red): 0.3 ± 0.03 g before and 0.11 ± 0.02 g during opto, *N* = 7]. (**D**) In SNI mice, optogenetic stimulation of 5-HT neurons induced a significant decrease in TL [total (black): 3.9 ± 0.22 s before and 2.9 ± 0.2 s during opto, *N =* 18; males (blue): 3.9 ± 0.24 s before and 2.9 ± 0.2 s during opto, *N* = 11; females (red): 3.9 ± 0.5 s before and 2.8 ± 0.33 s during opto, *N* = 7]. *P < 0.05 and ***P < 0.001, Wilcoxon matched-pair signed rank test.

**Fig. 8.**
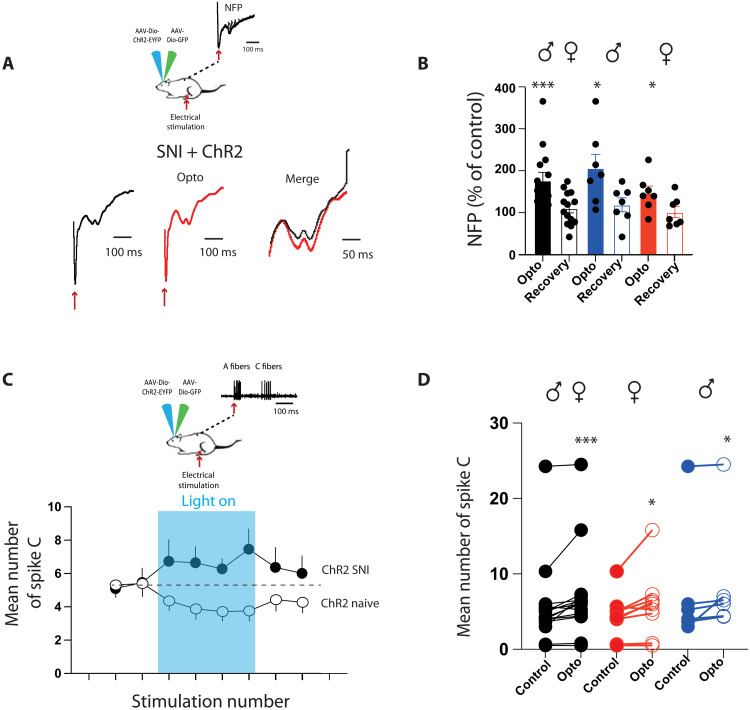
SNI alters dorsal horn neuron responses to 5-HT descending fiber activation. (**A**) In SNI mice, NFPs are increased upon optogenetic stimulation of 5-HT neurons. (**B**) On average, optogenetic stimulation increases NFP in both males and females [total (black): 176 ± 19.6% of control during opto, *N* = 14; males (blue): 206 ± 33% of control during opto, *N* = 7; females (red): 147 ± 17% of control during opto, *N* = 7]. *P < 0.05 and ***P < 0.001, one-sample *t* and Wilcoxon test. (**C**) In SNI mice, optogenetic stimulation of 5-HT neurons increases the response of WDR neurons to C-fibers. (**D**) Mean number of spike C is increased during optogenetic stimulation of 5-HT neurons in both males and females [total (black): 5.7 ± 1.5 spike C before and 7 ± 1.5 spike C during opto, *N* = 15; females (red): 4.4 ± 0.96 spike C before and 5.9 ± 1.5 spike C during opto, *N* = 9; males (blue): 7.7 ± 3.3 spike C before and 8.8 ± 3.1 spike C during opto, *N* = 6]. *P < 0.05 and ***P < 0.001, Wilcoxon matched-pair signed rank test.

**Fig. 9.**
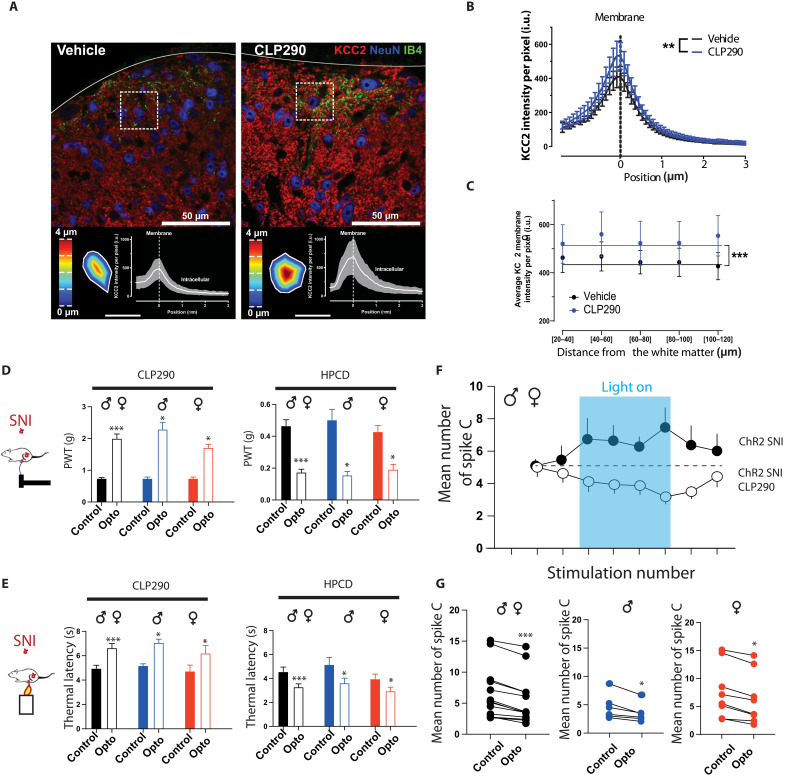
Chloride imbalance mediates the alteration of 5-HT descending fiber activation. (**A**) Confocal images showing KCC2 immunostaining in SNI mice treated with either vehicle or CLP290. Bottom panels show KCC2 expression profile in NeuN(+) cells. (**B**) Averaged intensity of KCC2 profiles of SNI + vehicle (black curve) and SNI + CLP290 (blue curve) neurons. (**C**) Average intensity in DHSC in SNI treated with either vehicle (black) or CLP290 (blue). (**D**) Under CLP290, stimulation of 5-HT neurons induced an increase in PWT in both males and females in SNI mice [total (black): 0.47 ± 0.04 g before and 2 ± 0.14 g during opto, *N* = 12]. Under HPCD (vehicle), the same stimulation reduced PWT in both male and female SNI mice [total (black): 0.5 ± 0.07 g before and 0.16 ± 0.02 g during opto, *N* = 12]. (**E**) Under CLP290, optogenetic stimulation of 5-HT neurons induced an increase in TL in both males and females in SNI mice [total (black): 5 ± 0.3 s before and 6.6 ± 0.36 s during opto, *N* = 12]. Under HPCD (vehicle), the same stimulation reduced TL in both male and female SNI mice [total (black): 4.6 ± 0.4 s before and 3.3 ± 0.3 s during opto, *N* = 12]. (**F**) Under CLP290, optogenetic stimulation of 5-HT neurons decreases WDR neuron response to C-fiber stimulations. (**G**) Mean number of spike C is decreased upon optogenetic stimulation of 5-HT neurons in both males and females [total (black): 6.6 ± 1.2 spike C before and 5.3 ± 1 spike C during opto, *N* = 14]. *P < 0.05 and ***P < 0.001, Wilcoxon matched-pair signed rank test.

We then studied the role of the 5-HT descending pathway in a pathological context using the spared nerve injury (SNI) model of painful mononeuropathy (*36*). We first confirmed that 2 weeks after SNI, mice expressed a strong mechanical and thermal hypersensitivity (Fig. 7, A and B) that was equivalent in both males and females. Using *epet::cre-Ai9::Tdtomato,* we first confirmed that 5-HT descending fibers are not modified in SNI as their density is equivalent to the one quantified in naïve animals, and their contacts are mostly on inhibitory interneurons again as in naïve animals (fig. S6). Therefore, we used *epet::cre* SNI mice to manipulate 5-HT descending fibers from the RMg spinal optogenetic stimulation of RMg 5-HT fibers to induce a reinforcement of mechanical and thermal hypersensitivity in both males and females. PWT and TL were lowered during optogenetic stimulation of 5-HT fibers (Fig. 7, C and D). In vivo electrophysiological recordings showed that these mechanical and thermal hypersensitivities were associated with an increased responsiveness of the DHSC (NFP; Fig. 8, A and B). Last, WDR neurons increased the number C-fiber spikes in response to peripheral stimulation during optogenetic activation of spinal RMg 5-HT fibers (Fig. 8C). These effects were equivalent in both males and females (Fig. 8D). These results strongly suggest that chloride imbalance in spinal neurons could modify descending pain controls by switching the impact of descending RMg 5-HT input from inhibition to facilitation. To address that possibility, we proposed to pharmacologically enhance KCC2 chloride transporters in SNI mice using the molecule CLP290 (*37*, *38*). We first confirmed that CLP290 induces an increase in membrane levels of KCC2. We performed immunohistochemical analysis on DHSC slices from SNI mice treated with either CLP290 or vehicle, and we found that the treatment in SNI mice (i) significantly increased KCC2 immunoreactivity in the spinal dorsal horn (fig. S7), (ii) significantly increased membrane KCC2 levels in individual dorsal horn neurons (Fig. 9, A to C), and (iii) induces a slight but significant increase in PWT and TL (fig. S7). Then, we compared pain behavior in SNI mice treated with either saline or CLP290 during optogenetic stimulation of 5-HT fibers (Fig. 9, D and E). In SNI mice treated with CLP290 but not vehicle, optogenetic stimulation of 5-HT fibers promoted mechanical and thermal analgesia as shown by a significant increase in PWT and TL in both males and females. Using in vivo electrophysiology recordings, we also found that, after CLP290 treatment, WDR neurons decreased their responsiveness to peripheral stimulations in both males and females upon optogenetic stimulation of RMg 5-HT fibers (Fig. 9, F and G). Together, these results show that KCC2-mediated chloride balance control descending influence: Normal chloride extrusion maintains effective RMg 5-HT descending input net inhibitory, while KCC2 hypofunction after nerve injury, causing chloride dysregulation, promotes RMg 5-HT descending input to become net facilitatory.

### Rescuing KCC2 function restores SSRI-mediated analgesia

**Fig. 10.**
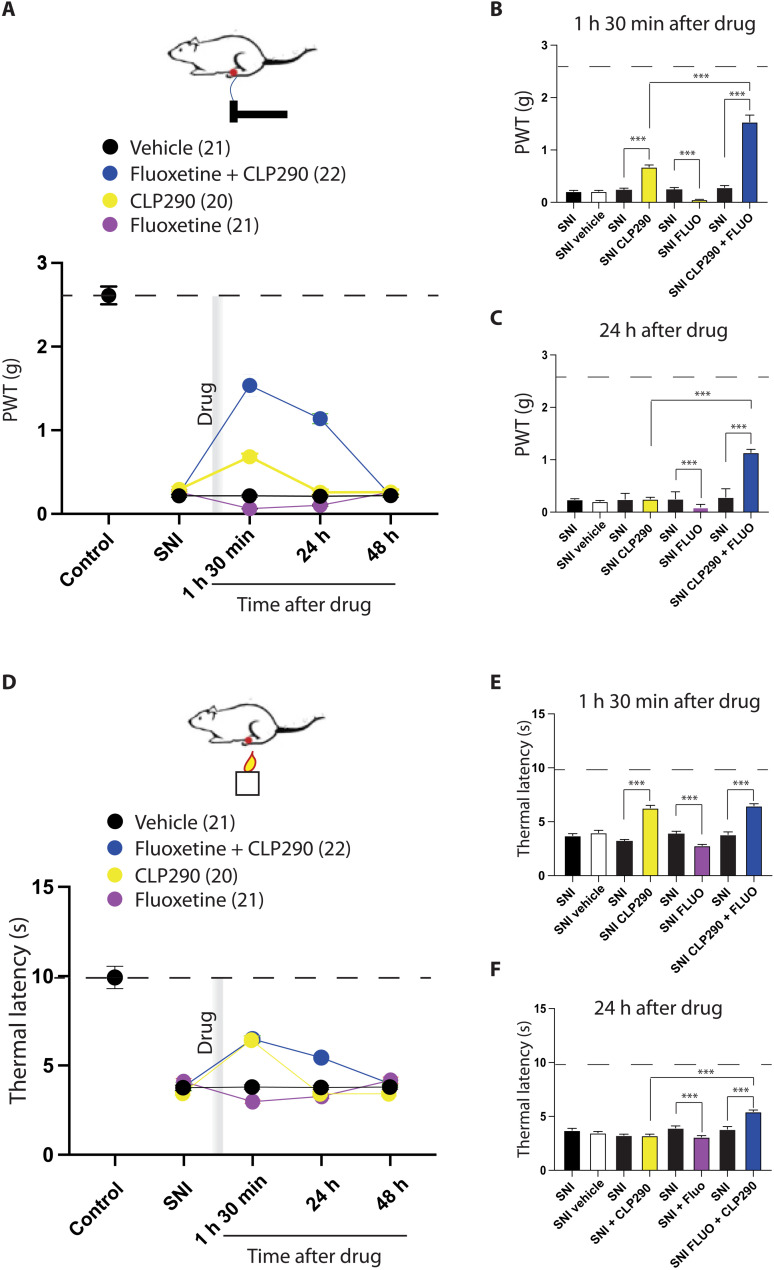
Combining SSRIs and KCC2 enhancers promotes pain relief in SNI mice. (**A**) PWT after a single injection of vehicle (black dots), fluoxetine (red dots), CLP290 (yellow dots), or the combination of fluoxetine (intraperitoneally) and CLP290 (per os, green dots). The combination induced a prolonged (>24 hours) and significant increase of PWT (*P* < 0.001, RM ANOVA, Dunnett’s multiple comparison test), while fluoxetine alone produces a prolonged hyperalgesia (*P* < 0.001, RM ANOVA, Dunnett’s multiple comparison test), and CLP290 induced a short and significant increase in PWT. (**B**) Comparison of each individual group value to predrug value, 1 hour 30 min after drug injection. (**C**) Comparison of each individual group value to predrug value, 24 hours after drug injection. (**D**) TL after a single injection of vehicle (black dots), fluoxetine (red dots), CLP290 (yellow dots), or the combination of fluoxetine (intraperitoneally) and CLP290 (per os, green dots). The combination induced a prolonged (>24 hours) and significant increase of TL (1 hour 30 min and 24 hours, *P* < 0.001, RM ANOVA, Dunnett’s multiple comparison test), while fluoxetine alone produces a prolonged decrease in TL (1 hour 30 min and 24 hours, *P* < 0.001, RM ANOVA, Dunnett’s multiple comparison test), and CLP290 induced a short and significant increase in TL (1 hour 30 min, *P* < 0.001, RM ANOVA, Dunnett’s multiple comparison test). (**E**) Comparison of each individual group value to predrug value, 1 hour 30 min after drug injection. (**F**) Comparison of each individual group value to predrug value, 24 hours after drug injection. The horizontal dotted line in graphs represents average control values for PWT and TL.

This mechanism may explain why SSRIs are not capable of relieving pain in patients with chronic pain (*18*, *39*). To test this hypothesis, we combined SSRI treatment with the KCC2 enhancer (Fig. 10). First, we confirmed in SNI mice that the SSRI fluoxetine injection alone induced a slight mechanical and thermal hypersensitivity (Fig. 10, in red). On the other hand, CLP290 induced a slight but significant decrease in mechanical and thermal hypersensitivity (Fig. 10, in yellow). By contrast, the combination of fluoxetine and CLP290 strongly relieved mechanical and thermal hypersensitivity (Fig. 10, in green). This analgesic effect was durable and still present 24 hours after a single injection of the combined molecules (Fig. 10, C and F). This effect was comparable in males and females (fig. S8). Last, to go beyond evoked pain responses that involve sensory-motor reflexes, we designed a conditioned place preference (CPP) paradigm to address the effect of our drug combination on supraspinal pain integration (fig. S9). SNI mice were first placed in a two-chamber maze of the same size but with an opaque and a transparent chamber and remained free to explore the whole maze (fig. S9A). Then, they were alternately locked in one or the other chamber; one was associated with either our drug combination (fluoxetine + CLP290; group 1) or fluoxetine alone (group 2). Two days after, mice were again left free to move in the maze and spent significantly more time in the chamber associated with the combination, but not in the chamber associated with fluoxetine alone (fig. S9B). These results show that SNI mice associate the chamber and the combination of fluoxetine and CLP and that this combination improves the painful state of the animal.

## DISCUSSION

These results show that 5-HT neurons of the RMg contact inhibitory interneurons in the DHSC and gate both mechanical and thermal nociceptive transmission in a sex-independent manner. Moreover, KCC2 down-regulation underlies the change in sign (i.e., excitatory or inhibitory) of 5-HT descending influence on nociceptive transmission, switching from pain inhibition to facilitation in the pathological condition (fig. S10). This result demonstrates that the balance between descending excitation and inhibition depends, contrary to previously thought, on local alteration at the spinal level. In particular, it suggests that the same 5-HT circuit can be excitatory and/or inhibitory depending on the excitability of the targeted region.

5-HT neurons are gathered in nine different nuclei of the brainstem called B1 to B9 (*40*, *41*). B1 to B3 nuclei project to the spinal cord, with B1 (i.e., raphe pallidus) and B2 (i.e., raphe obscurus) projecting to the ventral horn and B3 (i.e., medial RMg) and lateral nucleus paragigantocellularis (LPGi) projecting to the dorsal horn (*42*). Here, by crossbreeding *ePet::cre* mice to *Ai9::tdtomato* reporter mice, we confirmed the dorsal projection of 5-HT neurons in two preferential areas, superficial lamina and deep lamina. The former is attributed to the projection of the LPGi, and the latter is attributed to the projections of the nucleus raphe magnus (RMg) (*41*, *42*). Here, we focused on the medial RMg, and we confirmed the projections of 5-HT neurons from this nucleus to the deep layer of the spinal cord. Last, by using an AAV-dio-synaptophysin-myc, we show clear 5-HT terminals associated to inhibitory interneurons, suggesting that 5-HT release is synaptic rather than volumic in our circuit (*43*, *44*).

Optogenetic stimulation of 5-HT neurons of the medial RMg at a frequency slightly higher than the tonic rate of discharge of 5-HT neurons induces a specific increase in 5-HT levels in the spinal cord, in accordance with previous studies using electrical stimulation of the medial RMg (*45*, *46*). Associated with this increase, we observed a thermal and mechanical analgesia in naïve animals during light activation that ceased quickly after light stopped. This result is in contradiction with those obtained with a mouse constitutively expressing ChR2 in 5-HT neurons (*47*). In this study, optogenetic stimulation of 5-HT neurons induced a robust and long-lasting hypersensitivity to pain that lasts for several days following light illumination. Two differences may explain such discrepancy: First, we used a stimulation protocol that mimics the maximum rate of discharge of 5-HT neurons recorded in vivo (5 Hz/5 ms) in place of a high nonphysiological stimulation frequency (20 Hz/15 ms). A frequency-dependent mode of action of 5-HT neurons is in accordance with previous results that show that electric stimulation of the RMg can induce either pronociceptive or analgesic effects (*48*). Second, in the study from (*47*), they used a mouse that expressed ChR2 in all the B3 5-HT nuclei including the LPGi that is excluded from our study. Taking into account that 5-HT neurons from the LGPi project to the more superficial lamina of the dorsal horn, we cannot exclude that this circuit would be responsible for the pronociceptive effect observed, consequently suggesting two parallel descending pathways (one excitatory and one inhibitory) (*49*). This possibility is reinforced by recent studies showing that manipulation of different cortical circuits can give rise to either facilitatory or inhibitory 5-HT–dependent pathways controlling nociceptive transmission at the level of the spinal cord (*15*, *16*).

We show that 5-HT neurons from the medial RMg project to inhibitory interneurons in the DHSC. By blocking GABA_A_- and glycine receptor–mediated inhibitory neurotransmission with picrotoxin, we suppress the analgesic effect of the optogenetic stimulation of 5-HT neurons. 5-HT–induced neuronal responses are dependent on 5-HT receptors that comprise 7 families (5-HT1 to 5-HT7) and 15 subtypes. The more recent studies identified at least 12 subtypes involved in the dorsal horn response to 5-HT (*50*). Among them, at least three families can exert an action on inhibitory interneurons, i.e., 5-HT1, 5-HT2, and 5-HT3 (*3*, *51*). More precisely, 5-HT2a, 5-HT2c, and 5-HT3 receptors can increase inhibitory neurotransmission by a direct modulation of inhibitory terminals in both GABA_A_- and glycine receptor-mediated inhibitory neurotransmission (*30*, *31*, *51*). Activation of these receptors with specific agonists is also capable of generating a significant inhibition of the C-fiber component of dorsal horn neurons, and antagonists suppress the inhibitory effect of 5-HT application on the same neurons (*52*). Last, 5-HT2 receptor modulators including 5-HT2c agonists suppress mechanical allodynia in neuropathic pain models (*53*, *54*). Here, optogenetic manipulation of 5-HT neurons induced a mechanical and thermal analgesia associated with a decrease in the C-fiber component of the nociceptive receptive field and dorsal horn neurons in accordance with the strong inhibition of these neurons induced by RMg stimulation or 5-HT spinal application (*52*). Therefore, our results suggest that 5-HT neurons from the RMg exert a 5-HT2– and/or 5-HT3–mediated activation of local spinal inhibitory interneurons that block nociceptive inputs by decreasing excitability of dorsal horn neurons.

We used the SNI model of neuropathy to study the alteration of the RMg to the spinal cord circuit under pathological conditions. Alteration of 5-HT modulation in chronic pain is well documented in preclinical models. For instance, the influence of 5-HT3 receptor–mediated hyperexcitability is well known following peripheral nerve injury (*55*, *56*). Here, we show that RMg to spinal cord circuit is not modified in the SNI model of neuropathic pain. 5-HT fiber density is not altered in the DHSC of SNI animals, and 5-HT fibers target inhibitory interneurons in the DHSC as shown in naïve animals. However, optogenetic stimulation of the same circuit in SNI induces mechanical and thermal hyperalgesia, transforming a descending inhibition circuit into descending facilitation. We also show that this switch is a consequence of chloride imbalance following neuropathy. Chloride imbalance is a key feature in chronic pain models, particularly following peripheral neuropathy (*33*, *57*, *58*). Mechanistically, it is mediated by the decreased expression of the KCC2 cotransporter that normally mediates chloride outflux to maintain low intracellular chloride. KCC2 hypofunction induces a depolarizing shift in chloride equilibrium, yielding a weakening and sometimes even an inversion of GABA_A_ and glycine receptor-mediated inhibition (*59*). Whether the switch from net inhibition to net excitation reflects an inversion of the sign of GABA_A_ or glycine receptor–mediated transmission or the loss of balance within a mixture of excitatory/inhibitory effect of activation of descending 5-HT–mediated transmission remains to be determined. However, the critical finding is that manipulating KCC2 function with specific modulators (*37*, *60*) effectively switched the net effect of stimulation of the RMg-spinal 5-HT circuit: Inhibiting KCC2 promotes 5-HT descending net facilitation in naïve animals, and enhancing KCC2 promotes 5-HT descending net inhibition in neuropathic animals. Overall, this paved the way to a mechanistic handle to reverse a maladaptive loss of endogenous pain control, which can explain abnormal pain hypersensitivity in neuropathic pain syndromes.

Antidepressants are currently one of the first lines of medication to manage chronic pain in humans (*18*). Among the antidepressant family, SNRIs and tricyclic antidepressants (TCA) are the more efficient molecules but with moderate efficiency (relief of 50% of pain in approximately half of patients) and many side effects (*19*). SSRIs, by contrast, have little or no effect on pathological pain sensation but with fewer side effects, effectively singling out serotoninergic transmission as a viable target for analgesia (*39*). Here, we confirm that treatment with fluoxetine does not improve thermal or mechanical hypersensitivity, but if fluoxetine is associated with a KCC2 enhancer, it durably relieves pain hypersensitivity for at least 24 hours. Our findings posit that fluoxetine increases spinal 5-HT that activates inhibitory interneurons. Its ineffectiveness is attributed to spinal chloride dysregulation, causing it to promote pain facilitation after nerve injury. Restoring chloride extrusion in spinal neurons was sufficient to yield a net inhibitory action of fluoxetine in the spinal circuits that had undergone maladaptive reorganization in a pathological setting. By choosing to very selectively stimulate the RMg 5-HT pathway, isolating a pure descending serotonergic system, we offered a paradigm that enabled uncovering a mechanistic explanation for the failure of SSRI to produce analgesia in neuropathic pain. Given the lower side effect liability of SSRIs, the current findings open entirely new therapeutic perspectives, including repurposing for a class of drugs that is readily available for use in multiple other conditions.

To conclude, we identify a spinal mechanism responsible for the deficiency of endogenous pain control in pathological pain, which may explain the ineffectiveness of conventional treatments targeting these pathways. Therefore, combining SSRIs with chloride extruders presents a new therapeutic approach that will restore CPM in chronic pain syndromes, with the vital advantage of reempowering patients by reestablishing their ability to control their own pain.

## MATERIALS AND METHODS

### Animals

Male and female mice, between 6 and 12 weeks of age, were maintained under a standard 12-hour light/12-hour dark cycle with food and water available ad libitum. All experiments followed European Union (Council directive 86/609EEC) and institutional guidelines for laboratory animal care and use. Institutional license for hosting animals was approved by the French Ethical Committee (APAFIS#3751). Male and female mice exhibit the same responses; thus, we pooled results from both sexes in all figures. We used different transgenic mice strains derived from C57BL6/J genetic background: *ePet::Cre* (strain 012712; RRID: IMSR_JAX:012712), *ePet::Cre*X*Ai9::tdtomato* (strain 007909, RRID: IMSR_JAX:007909), *Gad67::GFP*, and *ePet::Cre*X*Gad67::GFP*. *ePet::Cre*: It enables specific expression of light-activated opsins and molecular or fluorescent tags. *ePet::Cre*X*Ai9::tdtomato*: It allows the expression of a tomato fluorescent tag in cre-expressing cells, used to verify the expression of CRE in targeted neurons and, thus, 5-HT–expressing cells. *Gad67::GFP* (strain 007677, RRID: IMSR_JAX:007677): Mouse strain expressing GFP fluorescent tag in Gad67-positive neurons mainly GABA inhibitory neurons. *ePet::Cre*X*Gad67::GFP*: CRE expression in 5-HT neurons and GFP expression in GABA neurons. In each experimental group, mice were sex-, weight-, and age-balanced. Animals with no trace of viral infection in the RMg, those whose optic fibers were removed after implantation, those with no exaggerated pain behavior after SNI procedure, and those with a loss of body weight > 15% were excluded from the study.

### Surgical procedure

We performed surgical procedures following (*61*).

#### Virus injection

For a functional specification of the role of 5-HT neurons of the RMg in the descending modulation of the nociceptive transmission, 6- to 10-week-old *ePet::Cre* male and female mice were deeply anesthetized under 4% isoflurane in an induction chamber, then moved, and maintained under 1.5% isoflurane. Eyes were protected with ocular gel (Ocry-gel, Lab TVM, France), 100 μl (0.3 μg/ml) of buprenorphine was intraperitoneally injected (and repeated 6 hours after the surgery), and 20 μl of lidocaine 1% was administered locally (scalp) to alleviate pain during surgery. Mice were placed into a stereotaxic frame (RWD Desktop Digital Stereotaxic Instruments, Shenzhen, China), and the head was fixed with ear bars and a feedback-controlled heating pad (World Precision Instrument, Sarasota, USA), which ensured maintenance of core body temperature at 37°C. The skin was opened to expose the skull, and the surface was cleaned to expose the Bregma and Lambda. To specifically target the RMg of the RVM in the brainstem, three sites of injection were used, −5.6/0.0/−5.6, −5.8/0.0/−5.6, and −6.1/0.0/−5.7 (antero-posterior/medio-lateral/dorso-ventral, respectively), and a small hole (1-mm diameter) was drilled to expose the surface of the brain (RWD Microdrill, Shenzhen, China). Pulled borosilicate glass capillaries (Ringcaps, DURAN, Hirschmann Laborgeräte, Germany) were used to microinject a total of 300 nl of selected viruses (100 nl at each coordinate of interest, at a speed of 50 nl/min). We used AAV-EF1a-DIOhChR2(H134R)-EYFP-WPRE-pA (4.5 × 10^12^ vg/ml UNC Vector Core) for light activation or AAV-CAG-Flex-GFP (4.8 × 10^12^ vg/ml, UNC Vector Core) as control. We also used an AAV-DIO-Synaptophysin-myc for anatomical tracing of 5-HT synapses (*62*). Glass capillaries were kept in the injection site 5 min after injection and then retracted slowly over 3 min. The surgical sites were sutured and cleaned with betadine and treated with 1% lidocaine. Animals were kept on a heating pad and monitored until fully recovered (30 min) before being returned to their cage and continued to be group-housed after the procedure.

#### Optic fiber implantation

Optical cannulas including ceramic ferrule (AMS Technologies, Germany) in combination with a multimode optical fiber (Ø200μm, Thorlabs, NJ, USA) were implanted either at 300 μm above the RMg in the RVM or between vertebrae and spinal cord (*28*). Before the cannula implantation, the optical fibers were prepared and cleaved to the appropriate length (5.5 mm for the RMg and <0.5 mm for the lumbar vertebrae), and the light intensity emitted through the fibers was assessed using a power meter (PM-100D, Thorlabs, NJ, USA) and set to 10 mW. For RMg implantation, the skin was opened to expose the skull, and the surface was cleaned to expose the Bregma and Lambda. A small hole was drilled to expose the surface of the brain. With the help of a stereotaxic cannula holder (Thorlabs, NJ, USA), the optical cannula was lowered into the hole, and dental cement was used to secure it in place. Once the cement was dry, it was covered with black nail varnish to reduce the risk of external illumination. For spinal cord implantations, a 1- to 2-cm incision was made slightly caudal to the peak of the dorsal hump to expose the lumbar spinal region. The vertebra of interest was identified, and then a small incision was made between the tendons and the vertebral column on either side. Vertebra was then secured using spinal adaptor clamps, and all tissues were removed from the surface of the bone. A small hole was drilled approximately 1 mm from the midline on either the left or right side. We positioned the optical fiber in the hole and used a small amount of superglue around the drilled hole and over the surface of the bone to reduce the possibility of bone bleeds and to secure the cannula in place. Afterward, we cemented the cannula in place using dental cement, and we sutured and cleaned the skin surrounding the dental cement securing the cannula implantation. Mice were weighed and placed on a heating blanket to awaken them, and they were allowed to recover before they were returned to their cage and continued to be group-housed after the procedure. An optogenetic stimulation protocol has been set according to the spontaneous discharge of 5-HT neurons measured in previous studies. On average, 5-HT neurons have a discharge of 1 to 3 Hz (*63*); we set the stimulation protocol at 5 Hz/5 ms to be sure to activate 5-HT neurons and elicit action potentials at each optogenetic stimulation (see Fig. 1C).

#### SNI model

The day before the surgery, mice were tested for their mechanical threshold as described below (see the “Pain behavior” section). Then, on the day of the surgery, mice were anesthetized under 2% isoflurane. A single skin incision was made into the mid-thigh level, and the biceps femoris muscles were separated by blunt dissection to expose the sciatic nerve and its three main branches. The common peroneal and the tibial nerves were ligated using 6-0 silk and cut with small scissors, removing a 2- to 4-mm piece of each distal nerve stump. The sural nerve was kept intact. The muscle and skin were then sutured using 6-0 silk. Mice were allowed to recover on a warmed surgical pad until they were able to move freely.

### Pain behavior

Behavioral experiments were carried out blind and performed following procedures previously described in (*61*). For optogenetic experiments, the experimenter did not know the virus (ChR2 or GFP) injected into the tested mice. For pharmacology, the experimenter was not aware whether the drug or vehicle was injected.

#### Paw withdrawal threshold

Animals were placed in a square plastic frame with a mesh grid on the floor. After 10 min of habituation, animals were tested for mechanical threshold using Von Frey Hairs (Bioseb, France). Five successive tests were performed by applying a Von Frey Hair on the plantar surface of the paw in mice remaining on their four paws. We assessed the mechanical threshold using the simplified up and down (SUDO) technique (*64*). Briefly, starting from the 10th filament (2 g), the response of the animal to the stimulation (withdrawal or not) decided the value of the next filament. The lower filament tested whether the animal withdrew the paw, and the upper filament tested the other case. Five successive filaments were applied. Each test was separated by around 20 s. A series of SUDO was performed before, during, and after optogenetic stimulation of 5-HT neurons of the RMg or spinal cord. PWT was evaluated using the last filament value +/−0.5 depending on the value of the fourth filament. To determine the value in grams, the algorithm PWT = 10^(x*F+*B*)^ was used. *F* is the filament number obtained using SUDO, and *x* and *B* were determined from a linear regression of the logarithm of the empirically measured filament bending force plotted against the filament number [the following equation was used: log (bending force) = *x* * filament number + *B*, with *x* = 0.182 and *B* = −1.47 when 7 < *F* < 14, and *x* = 0.240 and *B* = −2 when 2 < *F* < 9] [see (*64*)].

#### Repetitive stimulations

To assess for analgesic effect, after determining the PWT, a suprathreshold filament was selected, and five repetitive tests were performed before, during, and after optogenetic stimulation of the 5-HT neurons. The number of paw thresholds was plotted as a percentage of the total number of tests (i.e., five). To test for hyperalgesia effects, the same procedure was applied but with the choice of a subthreshold filament.

#### Thermal latency

Animals were placed in a Plexiglas cage with a transparent glass floor. An infrared laser beam of calibrated value was applied on the plantar surface of the paw until the animal withdrew its paw. The value of the latency was measured in seconds. Two trials separated by at least 30 s to avoid sensitization were performed before, during, and after optogenetic stimulation of 5-HT neurons of the RMg. An average value of each trial was then compared between conditions. Laser intensity was adapted to the pathophysiological condition: infrared intensity (IR50) was used for naïve animals that give a mean withdraw latency around 5 to 10 s. IR30 was used in SNI to have the same range of responses.

#### Conditioned place preference

On day 1, SNI mice were free to move in a two-chamber maze (chamber size, 30 cm by 30 cm) separated by a corridor (10 cm) for 10 min. One chamber contained an opaque wall and one chamber contained a transparent wall (30 cm high each). On day 2, one group of mice (group 1) received an intraperitoneal dose of fluoxetine (10 mg/kg) and an oral dose of CLP290 (100 mg/kg). One hour and 30 min after injection, mice were placed and restrained in one chamber for 10 min (drug-paired). Group 2 received an intraperitoneal dose of fluoxetine (10 mg/kg), and mice were restrained in one chamber for 10 min (drug-paired). On day 4, mice were placed again in the maze and were free to visit all chambers. The percentage of total time spent in each chamber was measured and compared in each group between day 1 (predrug) and day 4 (test).

### In vivo electrophysiology

In vivo electrophysiology was performed following procedures previously described in (*61*). Mice were anesthetized with urethane 20% and placed on a stereotaxic frame (M2E, Asnières, France). A laminectomy was performed on lumbar vertebrae L1 to L3, and segments L4 to L5 of the spinal cord were exposed. Extracellular recordings of WDR dorsal horn neurons were made with borosilicate glass capillaries (2 megohms, filled with 684 mM NaCl) (Harvard Apparatus, Cambridge, MA, USA). For NFPs, the signal was low pass–filtered. For single-unit recordings, the signal was high pass–filtered, and the criterion for the selection of a neuron was the presence of an A-fiber response (0 to 80 ms) followed by a C-fiber response (80 to 300 ms) to electrical stimulations of the ipsilateral paw with bipolar subcutaneous stimulation electrodes. Threshold for C-fiber–evoked response was evaluated. Trains of electrical stimulation (at 0.067 Hz) at two times the threshold for C-fibers were performed before, during, and after optogenetic stimulation, with an optic fiber placed above the recording site.

### c-Fos experiments

*epet::cre* mice expressing ChR2 in the RMg (see above) were anesthetized with urethane 20% and placed on a stereotaxic frame (M2E, Asnières, France). Surgical procedure was performed as previously described in (*61*). Briefly, a laminectomy was performed on lumbar vertebrae L1 to L3, and segments L4 to L5 of the spinal cord were exposed. An optic fiber was placed above the spinal cord to allow light illumination of 5-HT descending fibers. After 1 hour of stable anesthesia, an optogenetic stimulation at 5 Hz/5 ms for 2 min was performed. One hour later, mice were quickly euthanized and perfused through the left ventricle with 4% (w/v) paraformaldehyde (PFA) in 0.1 M phosphate-buffered saline (PBS). Spinal cords were extracted. dissected out, post-fixed for 3 hours at 4°C in the same solution, then cryoprotected in a solution of 0.1 M PBS and 25% sucrose overnight, and stored at −80°C. c-Fos immunostaining with c-Fos antibodies (rabbit anti-c-Fos (1/500; 9F6, mAB Cell Signalling Technology 2250 S) was performed as below (see the “For molecular identifications of spinal network” section).

### High-Performance liquid chromatography

Tissue samples from optogenetically stimulated and control mice were deposited in previously weighed Eppendorf tubes and placed back in the freezer at −80°C. The tubes containing the samples were cautiously weighed again the day of the biochemical analysis, and 200 μl of perchloric acid (HClO_4_ 0.1 N, 4°C) was added. The samples (weighing approximately between 16 and 34 mg with no significant differences between the stimulated and the non-stimulated group) were homogenized with ultrasound for about 8 s and centrifuged at 13,000 rpm for 30 min at 4°C. A volume of 10 μl of the supernatant was injected into an HPLC system.

The tissue concentrations of monoamines were measured by HPLC coupled to electrochemical detection. The mobile phase of the HPLC system was composed of methanol (7%), NaH_2_PO_4_ (70 mM), triethylamine (100 μl/liter), EDTA (0.1 mM), and sodium octyl sulfate (100 mg/liter) diluted in deionized water (pH 4.2, adjusted with orthophosphoric acid) as previously reported (*65*). It was filtered (0.22 μm) before its installation in the system. The mobile phase was delivered through the HPLC column (Hypersyl, C18 at a flow rate of 1.2 ml/min using an HPLC pump (LC10Ad Vp, Shimadzu, France). The column was protected by a Brownlee-Newgard precolumn (RP-8, 15 × 3.2 mm, 7 μm; C.I.L.). The injection of the samples (10 μl) was carried out by a manual injection valve (Rheodyne, model 7725i, C.I.L., Sainte-Foy-La-Grande, France) equipped with a loop of 20 μl. The monoamines exit the column at different retention times (approximately NA: 3′38; DA: 7′43; 5-HT: 17′35) and passed into the coulometric detection cell (Cell 5011, ESA, Paris, France) equipped with two electrodes. The potential of these two electrodes was fixed via the coulometric detector (CoulochemII, ESA) at +350 mV (oxidation) and −270 mV (reduction), respectively. Only the signals from the oxidation channel were monitored. The coulometric detector was connected to a computer through an interface (Ulyss, Azur system, Toulouse, France). The calibration curves were performed once the peaks in a standard solution (1 ng/10 μl) were well separated in the chromatogram. The calibration curves were adapted to the expected and heterogeneous quantities of monoamines (0.1 to 1 ng/10 μl for 5-HT and NA; 0.01 to 0.1 ng/10 μl for DA) (*66*). Using a timeline method, the gains used ranged from 10 nA (for DA) to 200 nA (for NA and 5-HT). Standard solutions were used before each series of samples to verify the chromatographic conditions. The overall sensitivity for the compounds ranged from 2.4 pg/10 μl for DA to 7 pg/10 μl for 5-HT with a signal-to-noise ratio of 3:1.

### In vitro patch-clamp recordings

Patch-clamp recordings were performed following procedures previously described in (*61*), on brain slices from mice expressing either AAV-EF1a-DIOhChR2(H134R)-mCherry-WPRE-pA or AAV-CAG-Flex-GFP. Briefly, 8- to 12-week-old mice were intracardially perfused during euthanasia (exagon/lidocaine: 300/30 mg/kg, i.p.) with ice-cold *N*-methyl-d-glucamine (NMDG) solution [containing the following: 1.25 mM ascorbate, 3 mM Na-pyruvate, 2 mM thiourea, 93 mM NMDG, and 93 mM HCl 37% (pH 7.3 to 7.4); osmolarity: 305 to 310 mosM]. Brains were quickly removed, and 250-μm slices containing the RMg were prepared with a VT1100S Leica vibratome in ice-cold oxygenated NMDG solution before recovery for 12 to 15 min at 34°C in oxygenated NMDG solution. Slices were then transferred at room temperature into artificial cerebrospinal fluid (aCSF) solution [containing the following: 124 mM NaCl, 2.5 mM KCl, 1.25 mM NaH_2_PO_4_, 2 mM MgCl_2_, 2.5 CaCl_2_, 227 2.5 mM d-glucose, and 25 mM NaHCO_3_ (pH 7.3 to 7.4); osmolarity: 305 to 310 mosM] for at least 1 hour. Slices were placed in the recording chamber under a microscope (Nikon EF600) outfitted for fluorescence and IR (infrared)-DIC (digital image correlation) video microscopy and perfused with oxygenated aCSF at 2 to 3 ml/min. Viable RMg 5-HT neurons were visualized with a fluorescence video camera (Nikon). Borosilicate pipette (4 to 6 megohms; 1.5-mm optical density; Sutter Instrument) was filled with an intracellular solution (containing the following: 128 mM K gluconate, 20 mM NaCl, 1 mM MgCl_2_, 1 mM EGTA, 0.3 mM CaCl_2_, 2 mM Na_2_–adenosine 5′-triphosphate, 0.3 mM Na–guanosine 5′-triphosphate, 0.2 mM adenosine 3′,5′-monophosphate, and 10 mM Hepes; 280 to 290 mosM, pH 7.3 to 7.4). Recordings were made using a Multiclamp 700B amplifier, digitized using the Digidata 1440A interface, and acquired at 2 kHz using pClamp 10.5 software (Axon Instruments, Molecular Devices, Sunnyvale, CA). Pipette and cell capacitances were fully compensated, but junction potential was not corrected. RMg 5-HT neurons were recorded in whole-cell current-clamp mode. Brain slices expressing ChR2 or GFP were opto-stimulated at 470 nm (5 Hz, 5 ms pulse width) for 10 s every 30 s. Laser intensity was set at 5 mW/mm^2^.

### Drug injections

Drug injections were performed as previously described in (*61*).

#### Intraperitoneal injection

Intraperitoneal injections of furosemide (25 mg/kg) dissolved in NaOH 1 N with NaCl or vehicle (NaOH and NaCl) were performed 30 min before behavioral tests. Intraperitoneal injections of fluoxetine (10 mg/kg) dissolved in NaCl or vehicle (NaCl) were performed 1 hour 30 min before behavioral tests. Naïve *epet::cre* mice were maintained with one hand, and a 26-gauge needle connected to a 1-ml syringe was inserted in the intraperitoneal left side of the mice. A volume of 10 μl was injected.

#### Intrathecal injections

Intrathecal injection of 10 μl of picrotoxin (30 μM), Vu 0463271 (25 μM), or vehicle (0.03% of dimethyl sulfoxide in ddH_2_O) in *epet::cre* mice was done 20 min before the behavioral tests. Mice were maintained with one hand by the pelvic girdle, and a 27-gauge needle connected to a 10-μl Hamilton syringe was inserted in the subarachnoidal space between vertebrae L5 and L6.

#### Per os administration

Oral administration of CLP 290 (100 mg/kg) or vehicle (20% 2-hydroxypropyl-β-cyclodextrin) was done 1 hour 30 min before the behavioral tests. Mice were maintained with one hand, and a force-feeding needle connected to a 1-ml syringe was inserted into the throat to deliver 250 μl of the drug or vehicle.

### Immunohistochemistry

As already described in (*61*) and after completion of experiments, mice were euthanized with urethane and perfused through the left ventricle with 4% (w/v) PFA in 0.1 M PBS. Brains and spinal cords were extracted, dissected out, post-fixed for 3 hours at 4°C in the same solution, then cryoprotected in a solution of 0.1 M PBS and 25% sucrose overnight, and stored at −80°C.

For verification of both viral expression and location of optic fiber in RMg, a series of 20-μm thin slices containing the RMg were incubated free-floating in 0.1 M PBS containing Triton X-100 (0.3%), bovine serum albumin (1%; Sigma-Aldrich), and chicken anti-GFP antibody (1:1000; Averlabs) overnight at 4°C. After washing with 0.1 M PBS, secondary antibodies, such as Alexa Fluor 488–conjugated goat anti-chicken (1:500), were added in 0.1 M PBS for 2 hours at room temperature. Sections were finally viewed on epifluorescence microscope (axiophot2, Zeiss) fitted with a 20× dry objective and both 40× and 63× oil immersion 1.3 numerical aperture (NA) objective.

For molecular identifications of the spinal network, spinal cord sections were incubated free-floating in 0.1 M PBS containing Triton X-100 (0.3%), bovine serum albumin (1%; Sigma-Aldrich), guinea pig anti-TLX3 antibody (1:1000; a gift from T. Müller), goat anti-Pax2 antibody (1:300; Bio-techne), rabbit anti-Myc antibody (1:500; Euromedex), rabbit anti-TPH2 antibody (1:1000; Bio-techne), and rabbit anti-GAD65/67 (G5163, Sigma-Aldrich) in combination with chicken anti-GFP antibody (1:1000; Averlabs) overnight at 4°C. After washing with 0.1 M PBS, secondary antibodies were added in 0.1 M PBS for 2 hours at room temperature. Depending on the primary antibody species used, we used Alexa Fluor 568–conjugated goat anti-guinea pig (1:500, Molecular Probes), Alexa Fluor 568–conjugated donkey anti-goat (1:500; Thermo Fisher Scientific), Alexa Fluor 568– or 647–conjugated goat anti-rabbit (1:500; Invitrogen), and Alexa Fluor 568–conjugated goat anti-mouse (1:500; Invitrogen) associated with Alexa Fluor 488–conjugated goat anti-chicken (1:500; Thermo Fisher Scientific). For c-Fos experiments, rabbit anti–c-Fos (1:500; 9F6, mAB Cell Signalling Technology 2250 S) was combined with goat anti-Pax2 antibody (1:300; Bio-techne). c-Fos primary antibody was used for 24 hours, and then Alexa Fluor 488–conjugated goat anti-rabbit (1:500; Thermo Fisher Scientific) and Alexa Fluor 647–conjugated donkey anti-goat (1:500; Thermo Fisher Scientific) were used for c-Fos and Pax2, respectively.

Reactions using biotinylated antibodies were further incubated with Alexa 568–conjugated streptavidin (1:500; Molecular Probes) for 1 hour at room temperature. Sections were then mounted on gelatin-coated slides with DakoCytomation Fluorescent Mounting Medium (Dako SA) and finally viewed on a confocal microscope (Leica TCS SPE, Mannheim, Germany) fitted with a 20× dry objective and both 40× and 63× oil immersion 1.3 NA objective; confocal image stacks (0.75-μm steps) were acquired for each sample, and sequential acquisition was used to prevent cross-talk between Alexa 488 and Alexa 568. Three-dimensional (3D) reconstructions were performed using imaris software (Oxford Instruments, UK) from z-stack acquisitions. For 5-HT fiber contacts to Pax3- or Tlx3-positive cells, immunofluorescent 5-HT fiber and Tlx3 or Pax2 cell bodies were isolated. Each 5-HT fiber as close as half-diameter of each cell body was considered as a contact. The percentage of contact was evaluated as the ratio between cell bodies contacted by at least one 5-HT fiber, and the total amount of cell bodies was identified in one 3D image. Statistics were performed with three different 3D images of three different lumbar spinal slices from separate animals.

#### 5-HT fiber volume measurement

The volume of 5-HT fibers in the DHSC in control and SNI mice was measured using *epet::cre-Ai9::tdTomato* that express tomato in 5-HT neurons. Three control and three SNI mice were used, and ipsilateral DHSC was used to compare between control and SNI. Using ImageJ plugging “3D object counters,” the volume occupied by 5-HT fibers in the DHSC was measured as a ratio of the total volume of the selected area. Three sections in three animals were used to compare control and SNI mice.

#### KCC2 measurements

Transverse sections were cut at 25 μm on a vibratome Leica VT 1200S (Leica Microsystems). Sections were permeabilized in PBS (pH 7.4) with 0.2% Triton X-100 (PBS + T) for 10 min, washed twice in PBS, and incubated for 24 hours at 4°C in a mixture of primary antibodies containing a rabbit polyclonal anti-KCC2 antibody (1:1000; MilliporeSigma, catalog no. 07-432) and a chicken polyclonal anti-NeuN (1:1000; MilliporeSigma, catalog no. ABN91) antibody diluted in PBS + T containing 10% normal goat serum. After washing with PBS, the tissue was incubated for 2 hours at room temperature in a solution containing a mixture of IB4 (Isolectin B4)lectin (Alexa Fluor 488–conjugated IB4, 1:200; Invitrogen, catalog no. I21411), goat Cy3 anti-rabbit purified secondary antibody (1:500; Jackson ImmunoResearch Laboratories Inc., catalog no. 111-165-144), and goat Alexa 647 anti-chicken antibody (1:500; Invitrogen, catalog no. A-21449), all diluted in PBS + T (pH 7.4). Sections were mounted on superfrost gelatin-subbed slides (Fisherbrand, catalog no. 12-550-15), allowed to dry overnight at 4°C, and coverslipped using fluorescence mounting medium (Dako, catalog no. S3023). Confocal images were acquired using a Zeiss LSM 880 Confocal Laser Scanning Microscope. A plan-apochromat 20× M27 objective of numerical aperture 0.8 with a 0.7 zoom was used to localize the loss of IB4 staining due to SNI in dorsal horn sections. Low-magnification acquisitions were 12-bit images of 1560 × 1560 pixels with a pixel size of 0.389 μm and a pixel dwell time of 1.34 μs. Lines were averaged twice (fig. S8A). As KCC2 is absent from C-fiber afferents that contain IB4(+) terminals, the region of loss IB4 staining was used to quantify changes in KCC2 immunostainings at high magnification (*67*). An oil-immersion 63× plan-apochromatic objective of numerical aperture 1.40 was used for high-magnification CLSM (confocal laser scanning microscopy) images that were processed for quantification. High-magnification acquisitions were 12-bit images of 1560 × 1560 pixels with a pixel size of 0.087 μm and a pixel dwell time of 1.34 μs. Lines were averaged twice. Laser powers were adequately chosen to avoid saturation and limit photobleaching. All the acquisitions were performed with the same laser settings [i.e., laser, power, photomultiplier tube (PMT) settings, image size, pixel size, and scanning time]. During the acquisitions, the experimenter was blind to the slice conditions (i.e., vehicle versus CLP290 conditions).

### KCC2 measurements and analysis of subcellular distribution

The analysis method presented in this study is based on an already published algorithm used to quantify receptor membrane internalization (*68*). This technique was designed to reduce conscious or unconscious biases that can arise from user interventions. Before the analyses, the intensity of the immunostaining background noise was defined from white matter of the spinal dorsal horn, a region where KCC2 is known to be absent. For each image, the background average intensity was subtracted to the whole image to exclusively calculate the KCC2(+) immunostaining contribution. All neurons, identified by a NeuN(+) labeling, present in the immunocytochemistry confocal image were considered. The distance of each neuron from the gray/white matter border was first estimated. This provides for each neuron a measure of anatomical position in the dorsal horn and will be used for pooling KCC2 intensity profiles. To measure the subcellular KCC2 intensity profiles, the membrane regions of neuronal cells were delineated. For each pixel in the region of interest, the distance to the closest membrane segment was calculated. Using this distance map, the mean pixel intensity and SD of KCC2 fluorescence signal were quantified as a function of the distance to the neuronal membrane (defined as zero). Positive axis values correspond to neuronal intracellular space (Fig. 9, A and B). KCC2 subcellular intensity profiles were averaged as a function of the distance from gray/white matter border (Fig. 9C). The average pixel intensity profiles for each analyzed neuron were pooled together (distance bins from gray/white matter border of 20 μm, from 20 to 120 μm) to generate a graph of the KCC2 expression as a function of the anatomical position in the dorsal horn (Fig. 9G). A ratio paired *t* test was used to compare KCC2 membrane immunostaining intensities between vehicle and CLP290 at the different distance ranges from the gray matter surface. A total of 542 neurons (*N* = 6 mice) for the vehicle condition and 534 neurons (*N* = 8 mice) for the CLP290 condition were considered. The KCC2 membrane intensity profile for the 100- to 120-μm bin (68 neurons from 6 vehicle mice and 68 neurons from 8 CLP290 mice) was shown to illustrate the KCC2 membrane intensity profile in Fig. 2G. A ratio paired *t* test was used to compare KCC2 membrane immunostaining intensities between vehicle and CLP290 at 0, 1, 2, and 3 μm from the membrane.

### Statistical analysis

#### Behavioral analysis

Mechanical and thermal thresholds were compared before and during optogenetic stimulations. A Wilcoxon matched-pair signed rank test was performed to compare before and during optogenetic stimulation (*N* = number of mice tested). For pharmacological approach and CPP (Fig. 9 and fig. S9), comparison before and after drug application was performed using RM (Repeated Measures) analysis of variance (ANOVA) followed by Dunnett’s multiple comparison test. Comparisons between groups were performed using Mann-Whitney test.

#### Electrophysiology

NFPs were measured between 0.1 and 0.3 ms after stimulation, and the area under the curve was also measured. A mean curved area was evaluated as the average of curved areas after four successive stimulations. Mean NFPs during and after optogenetic stimulation was compared to the initial condition by measuring a percentage of change that was statistically assessed with one sample *t* test (*N* = number of NFPs tested). For single-unit recordings, we calculate the number of C-fiber–induced spikes of WDR neurons after each electrical stimulation, for four stimulations before and during optogenetic stimulation. A Wilcoxon matched-pair signed rank test was performed to compare before and during optogenetic stimulation (*N* = number of cells recorded).

## Notes

View/request a protocol for this paper from *Bio-protocol*.
