# Supplementary material for: Switch of serotonergic descending inhibition into facilitation by a spinal chloride imbalance in neuropathic pain
Source: Sci Adv. 2022 Jul 27;8(30):eabo0689. doi: 10.1126/sciadv.abo0689 (PMC9328683; doi:10.1126/sciadv.abo0689)
Supplement: Supplementary file 1 — Figs. S1 to S10 [file sciadv.abo0689_sm.pdf]

Science Advances
AAAS

Supplementary Materials for

Switch of serotonergic descending inhibition into facilitation by a spinal chloride imbalance in neuropathic pain

Franck Aby *et al.*

Corresponding author: Pascal Fossat, pascal.fossat@u-bordeaux.fr

Sci. Adv. **8**, eabo0689 (2022)
DOI: 10.1126/sciadv.abo0689

This PDF file includes:

Figs. S1 to S10

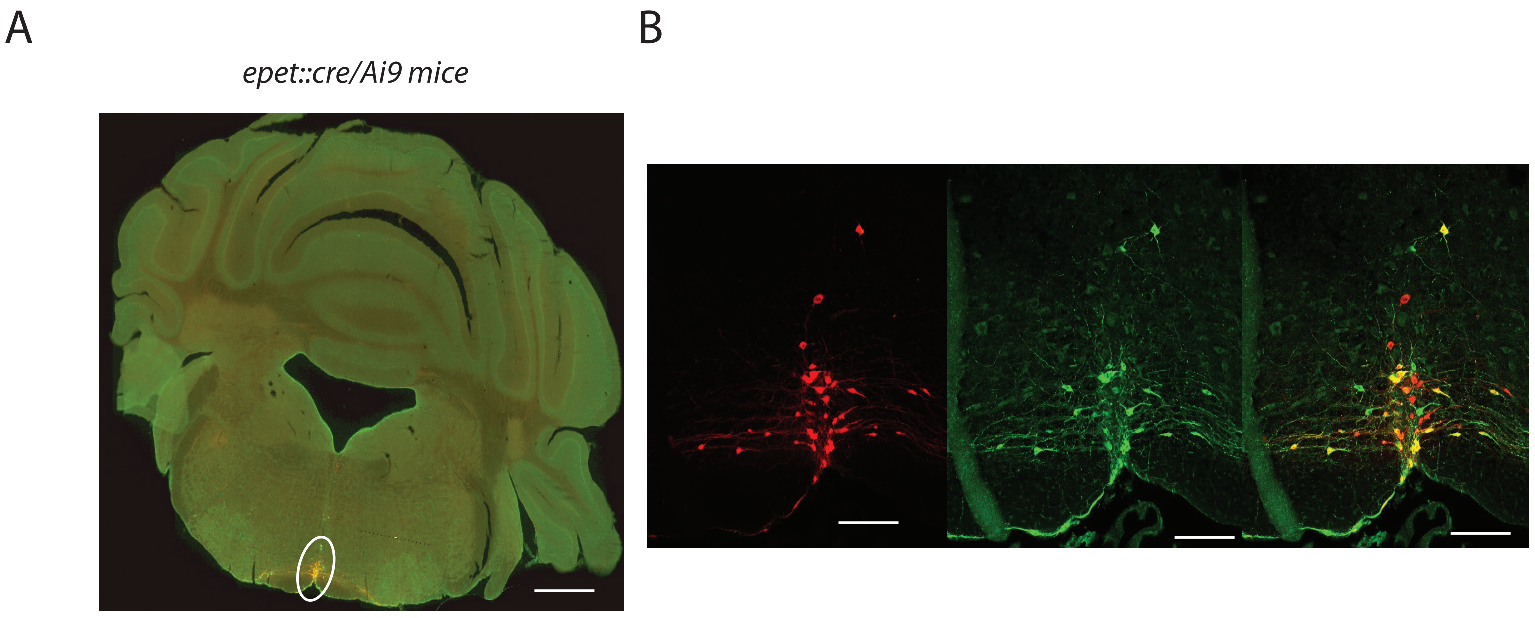

SupFig1 : Aby et al. 2022

In ePet::Cre mice, CRE recombinase expression was highly restricted to 5HT neurons. We crossbred ePet::Cre mice with an Ai9::t-dtomato reporter mice. A) Tomato staining in red and TPH2 (5HT biosynthesis enzyme) immunostaining in green in RMg of epet::cre/Ai9 mice. B) higher magnification showing that immunostaining against TPH2 is highly colocalized with tomato fluorescence. Tomato(+) cells were TPH2(+) at 90 2.3% and TPH2(+) cells were tomato(+) at 92,5 3.1%.

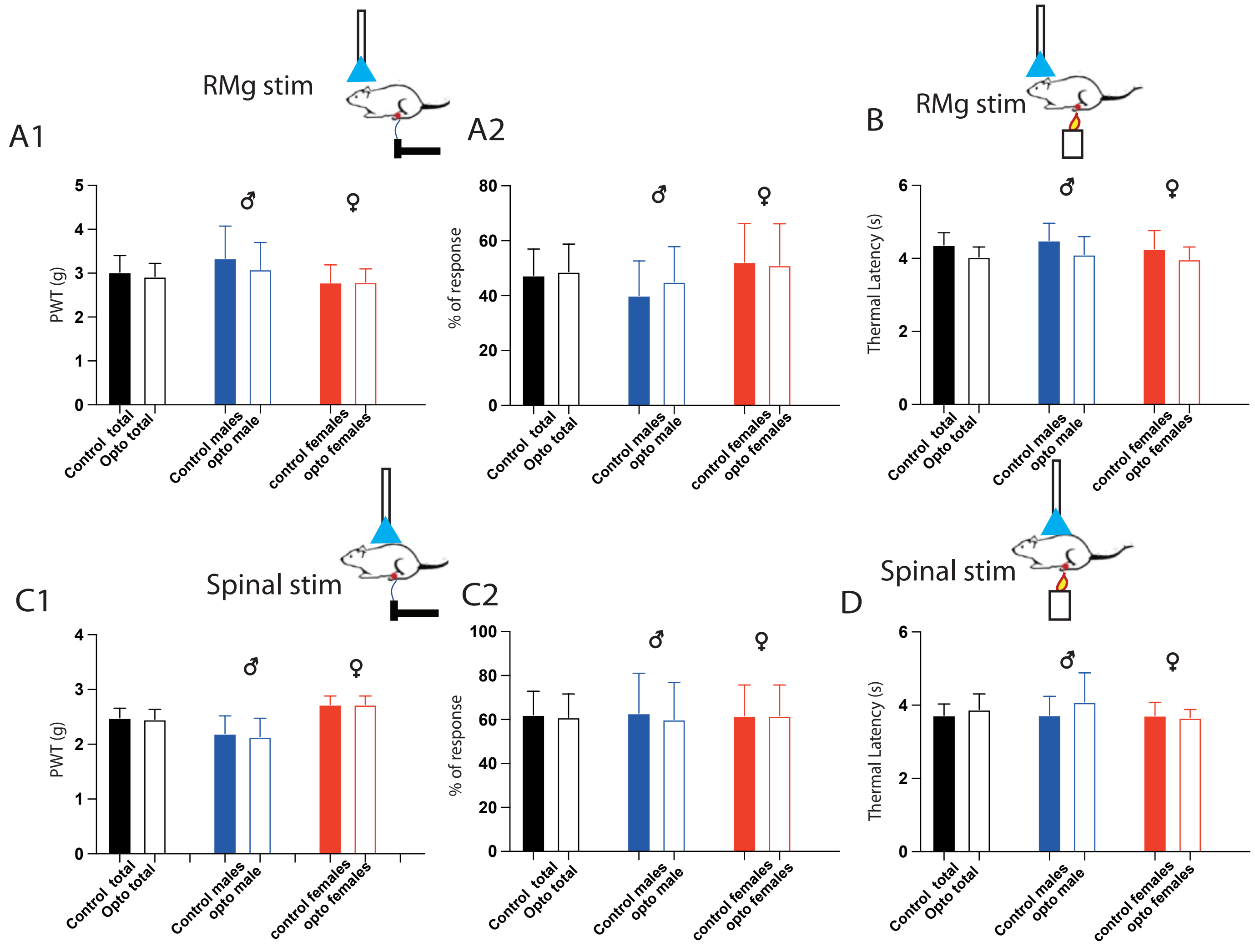

SupFig2 Aby et al 2022

Light illumination has no effect on PWT or TL. A) Mechanical response to von frey stimulation in ePet::Cre mice injected with a control AAV-dio-GFP (ChR2(-) mice). A1) PWT is not modified by light illumination of RMg in both males and females. A2) % of response to repetitive stimulation of the same VonFrey filament is not modified by light illumination of RMg. B) TL is not modified neither in males nor in females during light illumination of RMg. C-D) Same results as in A-B) obtained after light illumination of 5HT descending fibers.

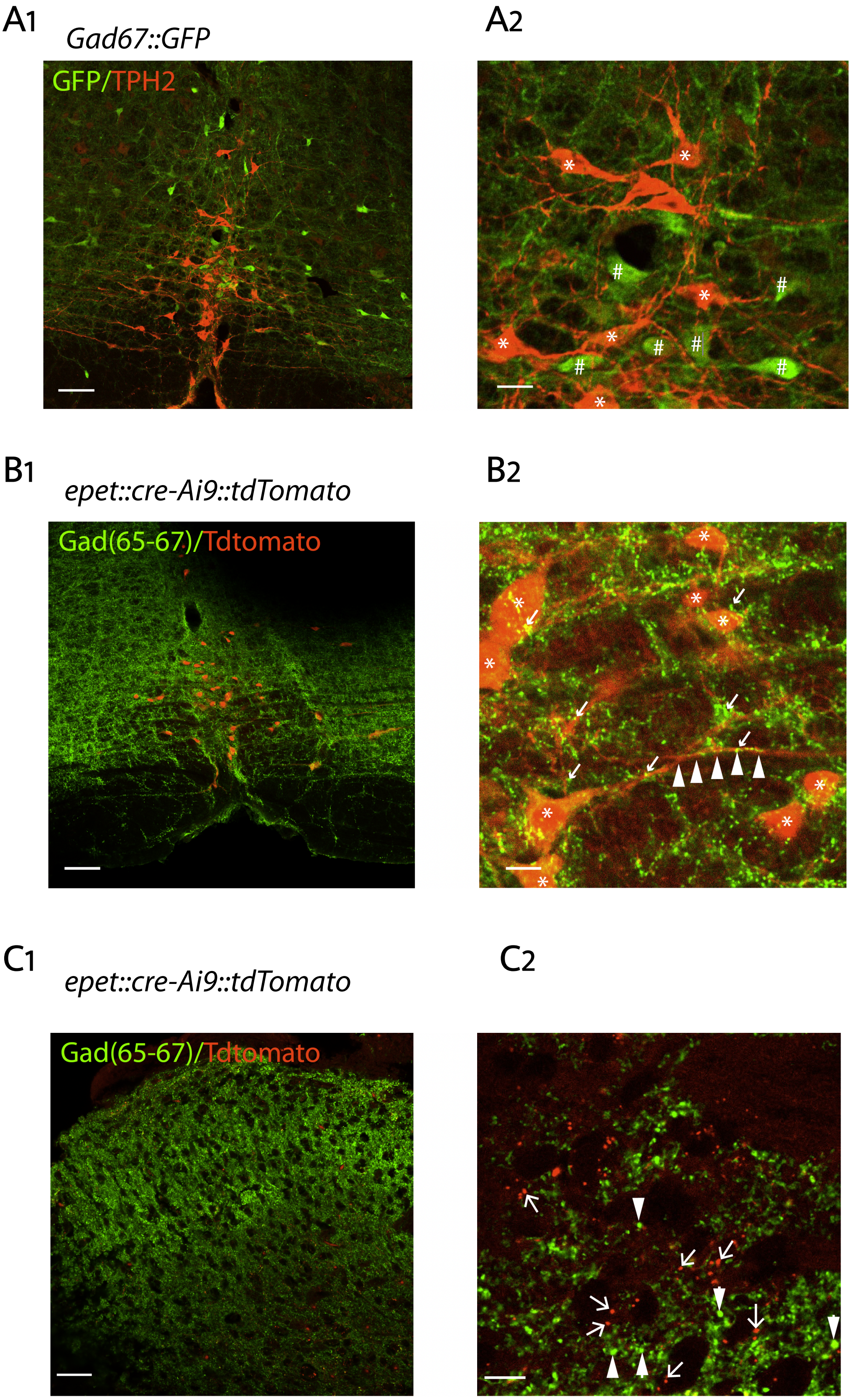

SupFig3. Aby et al, 2022

No colocalization between GABA and 5-HT. A1-2) In *Gad67::GFP* mice, TPH2 immunostaining and GFP staining are expressed in 2 separated neuronal population in the RMg (scale bar: 50 µm). B) Higher magnification (scale bar: 10µm). B1-2) In *epet::cre-Ai9::tdtomato* mice, tomato fluorescence show 5-HT neurons, and Gad65 immunostaining show GABA terminals in the RMg. 5-HT neurons are contacted by GABA terminals, but no 5-HT fibers are colocalized with GABA terminals excluding neurons with both GABA and 5-HT molecular identity. B2) Higher magnification. C1-2) In *epet::cre-Ai9::tdtomato* mice, tomato fluorescence show 5-HT neurons, and Gad65 immunostaining show GABA terminals in the DHSC. 5-HT and GABA terminals are not colocalized (scale bar 100µm). C2) Higher magnification (scale bar 10µm).

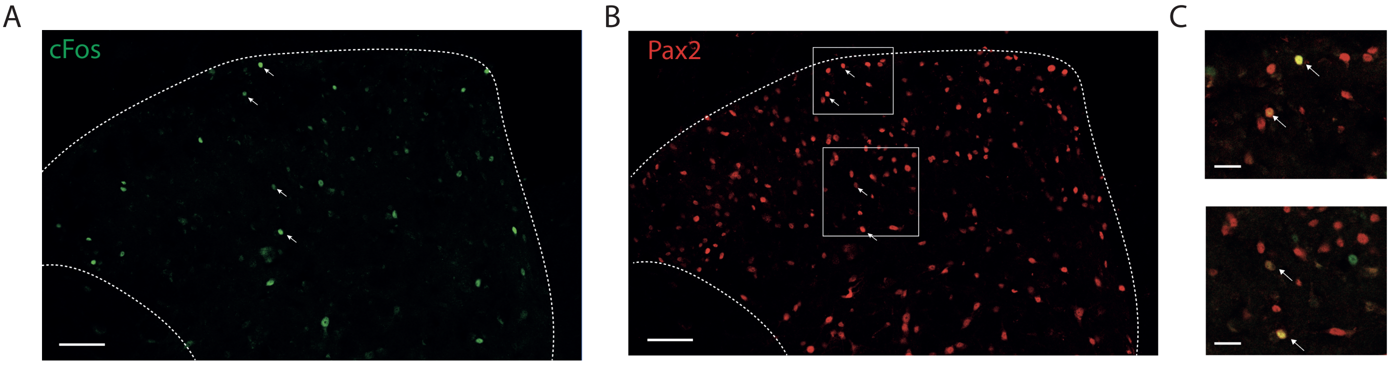

SupFig4. Aby et al, 2022

Optogenetic activation of ChR2 in 5-HT fibers induces cFos expression in inhibitory interneurons. A) cFos staining 1hr after optogenetic activation of 5-HT fibers. B) Pax2 immunostaining of the same section (scale bar: 50 µm). C) Higher magnification for superficial (upper panel) and deep dorsal horn layer (lower panel) showing cells with double labelling (scale bar:20 µm).

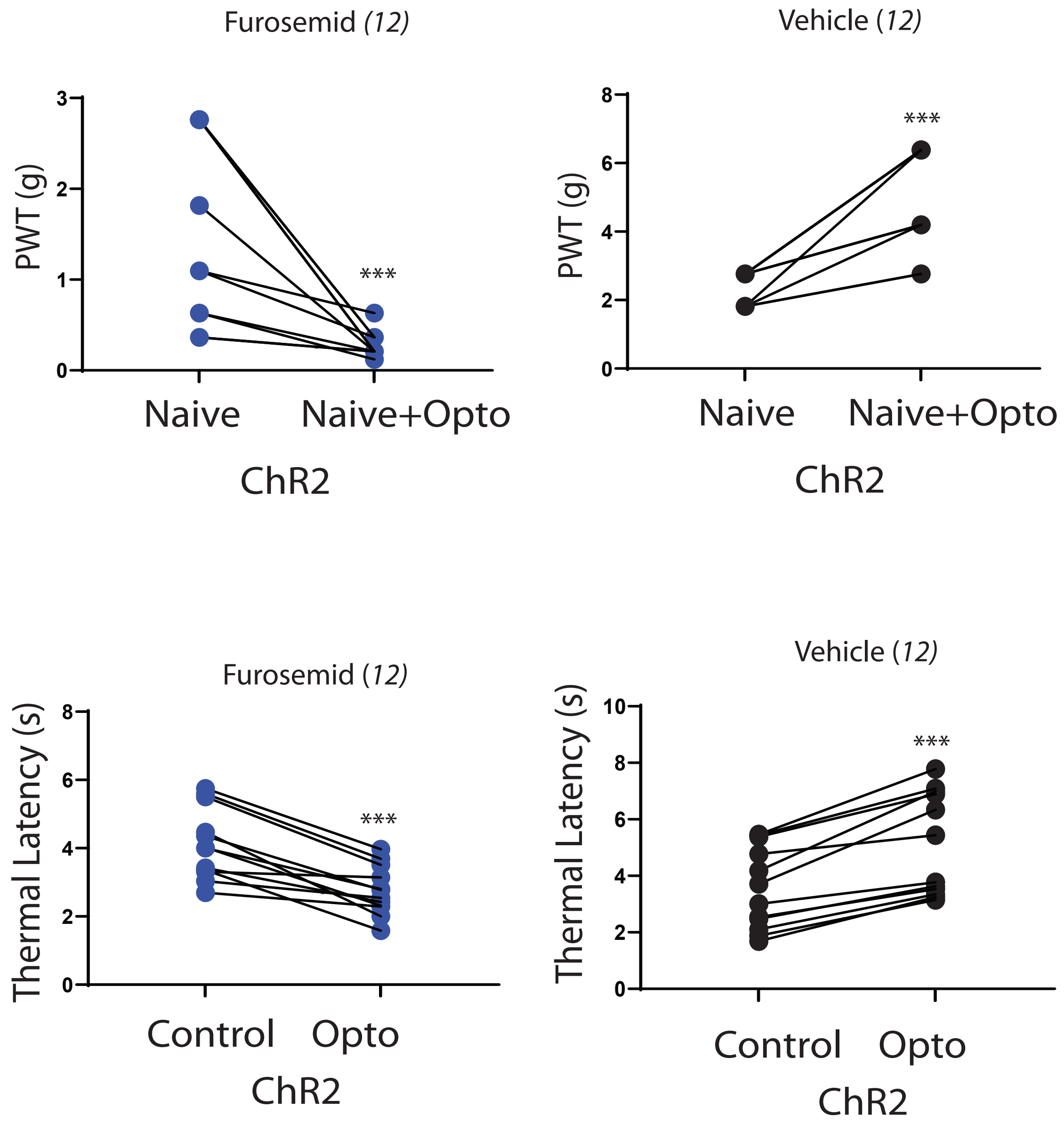

SupFig 5. Aby et al, 2022

Chloride transporter inhibition induces pain hypersensitivity. A) After ip injection of furosemid, subsequent activation of RMg neurons with ChR2 induces a decrease of mechanical threshold (1.7±0.2g before, 0.46±0.07g during optogenetic stimulation, $p<0.001$, n=12). B) After ip injection of vehicle, subsequent activation of RMg neurons with ChR2 induces an increase of mechanical threshold (2.5±0.09g before, 4.1±0.32g during optogenetic stimulation, $p<0.001$, n=12). C) After ip injection of furosemid, subsequent activation of RMg neurons with ChR2 induces a decrease of thermal latency (4.1±0.3s before, 2.8±0.2s during optogenetic stimulation, $p<0.001$, n=12). D) After ip injection of vehicle, subsequent activation of RMg neurons with ChR2 induces an increase of thermal latency (3.6±0.4s before, 5.1±0.5s during optogenetic stimulation, $p<0.001$, n=12). number of mice are in parentheses.

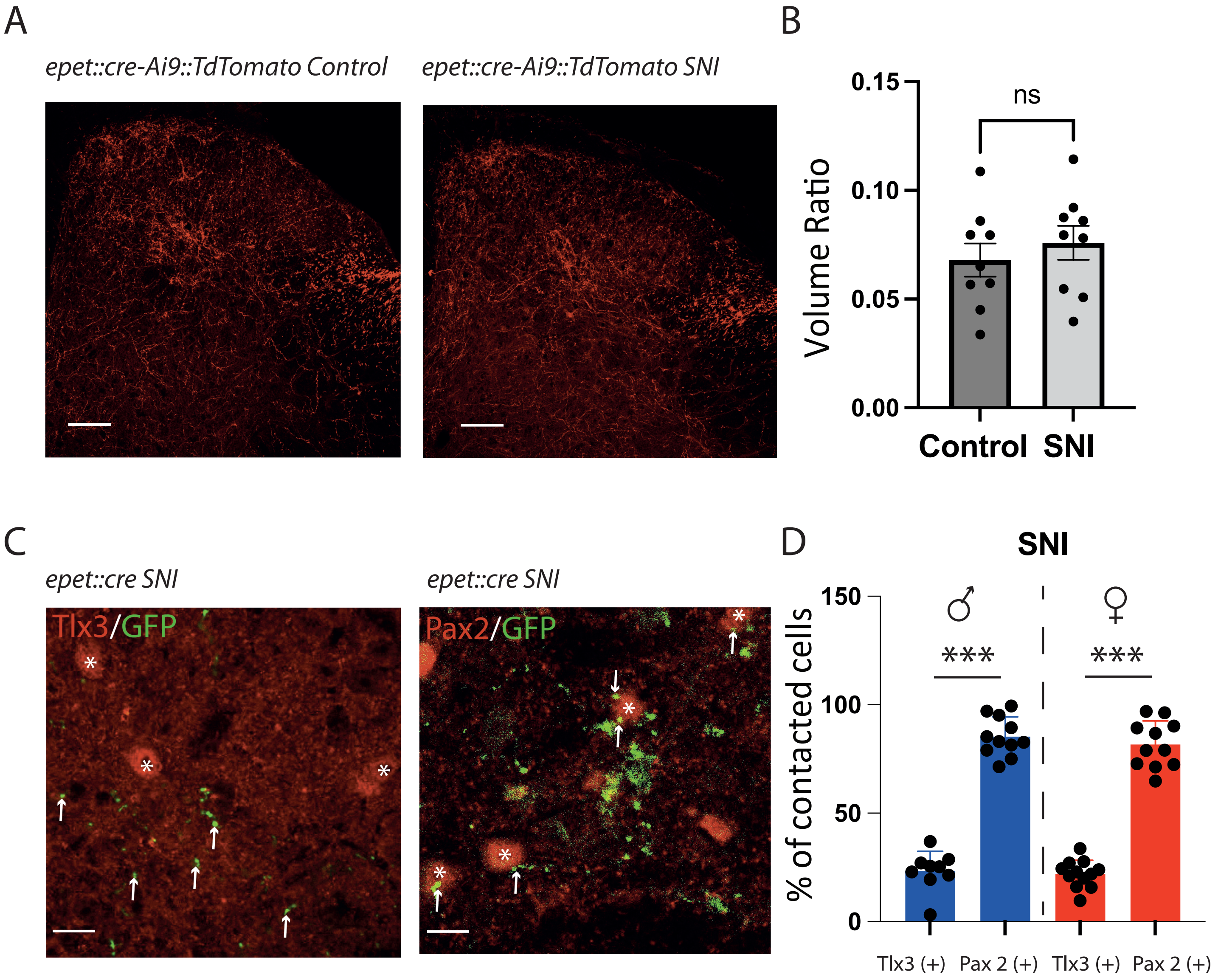

SupFig6 Aby et al, 2022

5-HT descending fibers are not altered in SNI mice. A) Dorsal horn slices with tomato fluorescence in epet::cre-Ai9::Tdtomato control (left) and SNI (right) mice (scale bar: 50 µm). B) 5-HT fibers occupy the same volume in the dorsal horn of the spinal cord in control and SNI mice (p=0,5, Mann-Whitney). C) Co-immunostaining for GFP with Tlx3 (C1) and Pax2(C2) in epet::cre SNI mice injected with an AAV-Flex-GFP (scale bar 20 µ m). D) Contacted cells are significantly higher for Pax2(+) than for Tlx3(+) both in SNI males and SNI females (males (blue): 85.4 2.7% for Pax2 and 23.4 3% for Tlx3, p<0,001, Mann Whitney; females (red): 81.7 3.3% for Pax2 and 22 1.8% for Tlx3, p<0.001, Mann Whitney).

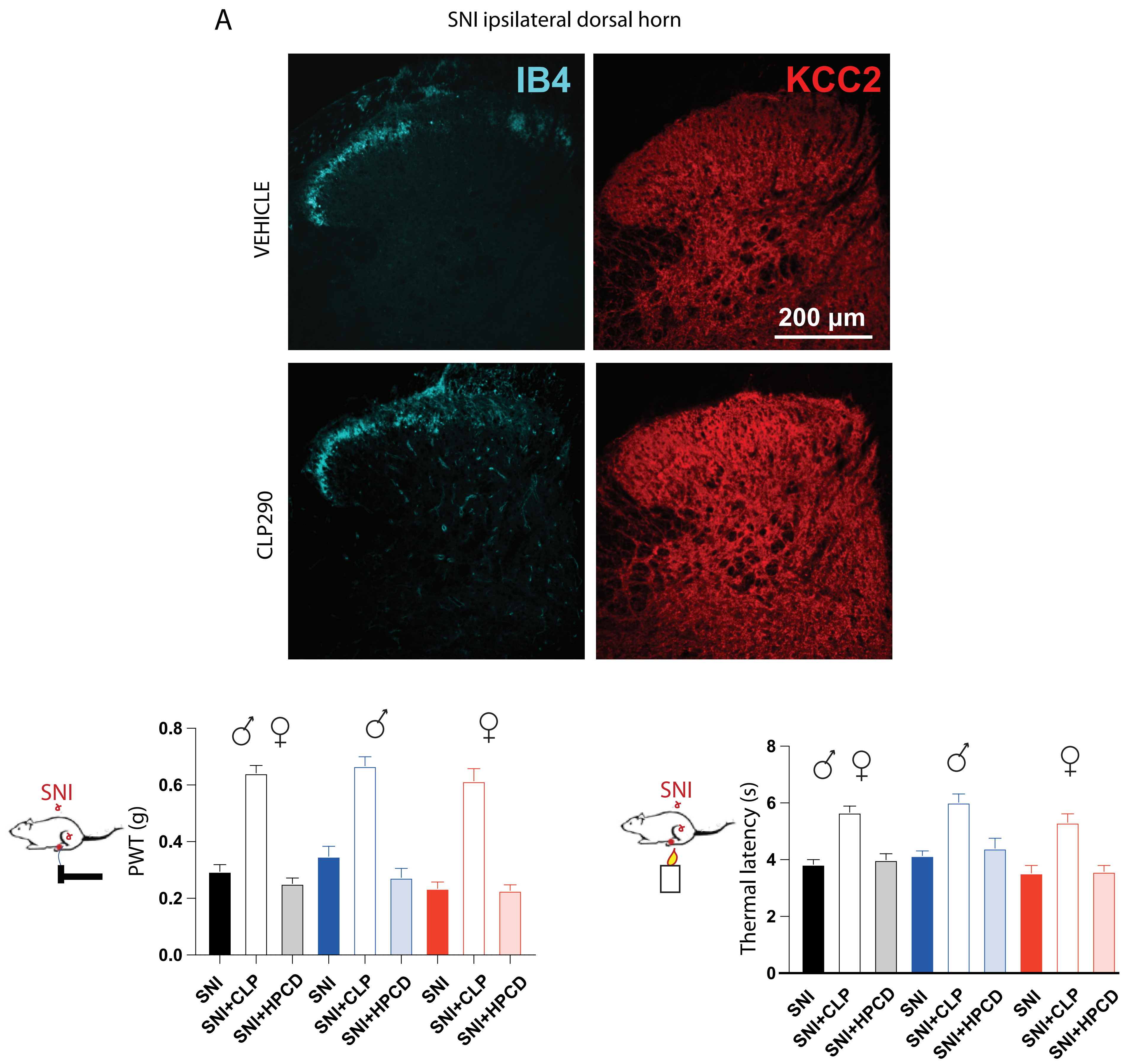

SupFig 7. Aby et al. 2022

CLP290 induces increase in KCC2 immunoreactivity in dorsal horn and partial mechanical and thermal pain relief. A) Left Panel, IB4 staining highlighting lamina II, right panel KCC2 staining. Upper panel after HPCD, lower panel after CLP290. Note the higher fluorescence intensity after CLP290 treatment. B) images at higher magnification in the IB4+ lamina. Note the higher dots fluorescence for KCC2 (in red) after CLP290 treatment. C) per os injection of CLP290 in SNI mice induces a significant increase in PWT in both males and females (Total (Black); 0.3±0.02g Before injection, 0.64±0.02g after CLP290 and 0.25±0.02g after vehicle, N=32. Males (Blue); 0.35±0.03g before, 0.67±0.03g after CLP290, N=17. Females (red); 0.23±0.02g before, 0.61±0.04g after CLP290, N=15). D) per os injection of CLP290 in SNI mice induces a significant increase in thermal latency not present after vehicle injection (Total (Black); 3.8±0.16s Before injection, 5.7±0.2s after CLP290 and 4±0.22s after vehicle, N=26. Males (Blue); 4.1±0.17s before 6±0.3s after CLP290, 4.4±0.35s after HPCD, N=13. Females (red); 3.5±0.3s before injection, 5.3±0.3s after CLP290, 3.6±0.2s after HPCD, N=13). **p<0.01, ***p<0.001 (RM ANOVA followed by Dunn’s post hoc test. Number of mice are in parentheses.

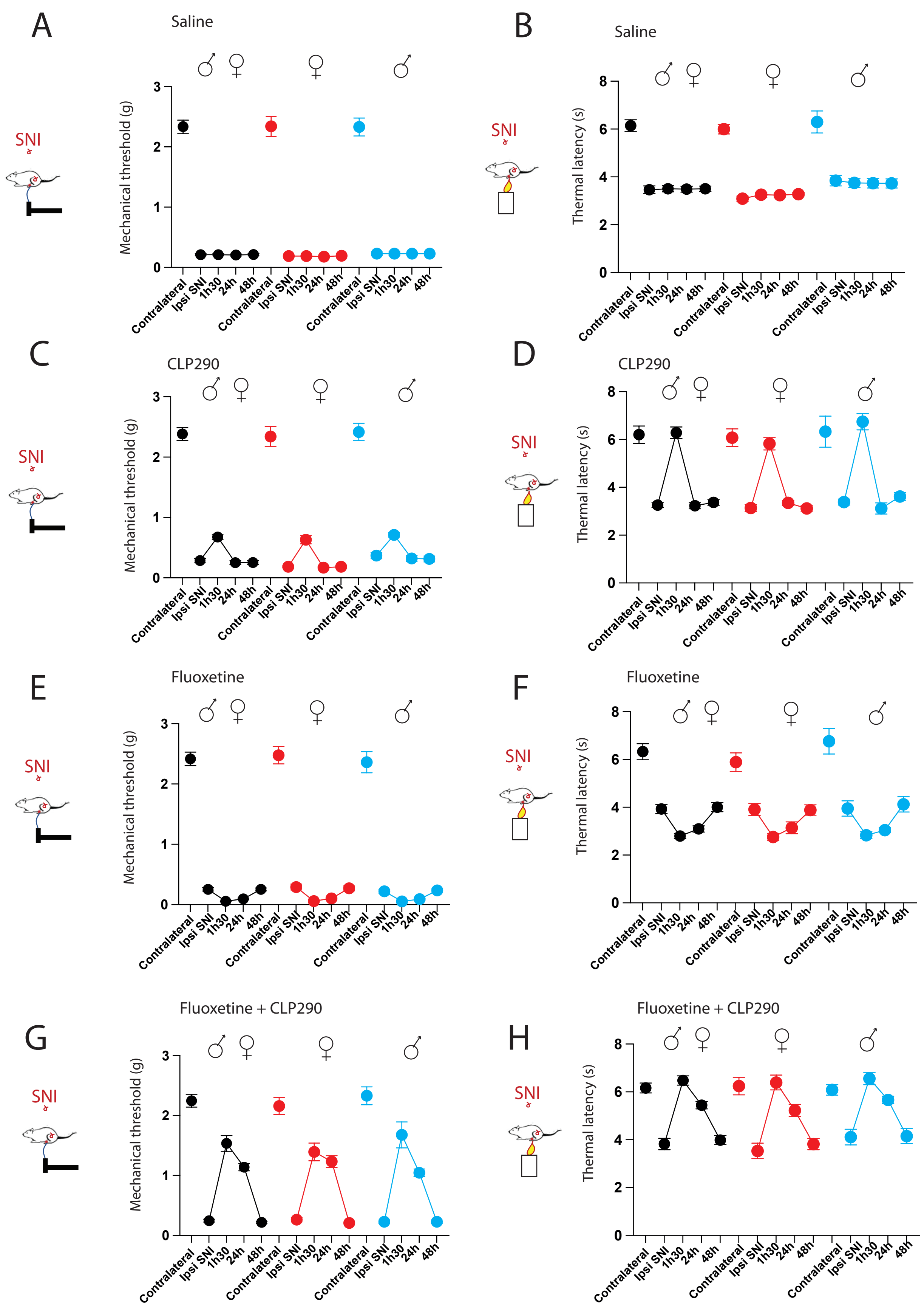

SupFig8 Aby et al, 2022

Combination of fluoxetine with CLP290 induces long lasting analgesia in SNI mice. A-B) Vehicle injection does not change PWT and TL in both male and female mice. C-D) CLP290 alone induces a slight but significant mechanical analgesia and a robust thermal analgesia in both male and female. Effect of CLP290 disappears 24hrs after injection. E-F) Fluoxetine injection worsen mechanical and thermal hypersensitivity in both male and female SNI mice. This effect is long lasting always present 24hrs after fluoxetine injection. G-H) Combination of fluoxetine and CLP290 induces a significant mechanical and thermal analgesia lasting for at least 24hours in both male and female mice.

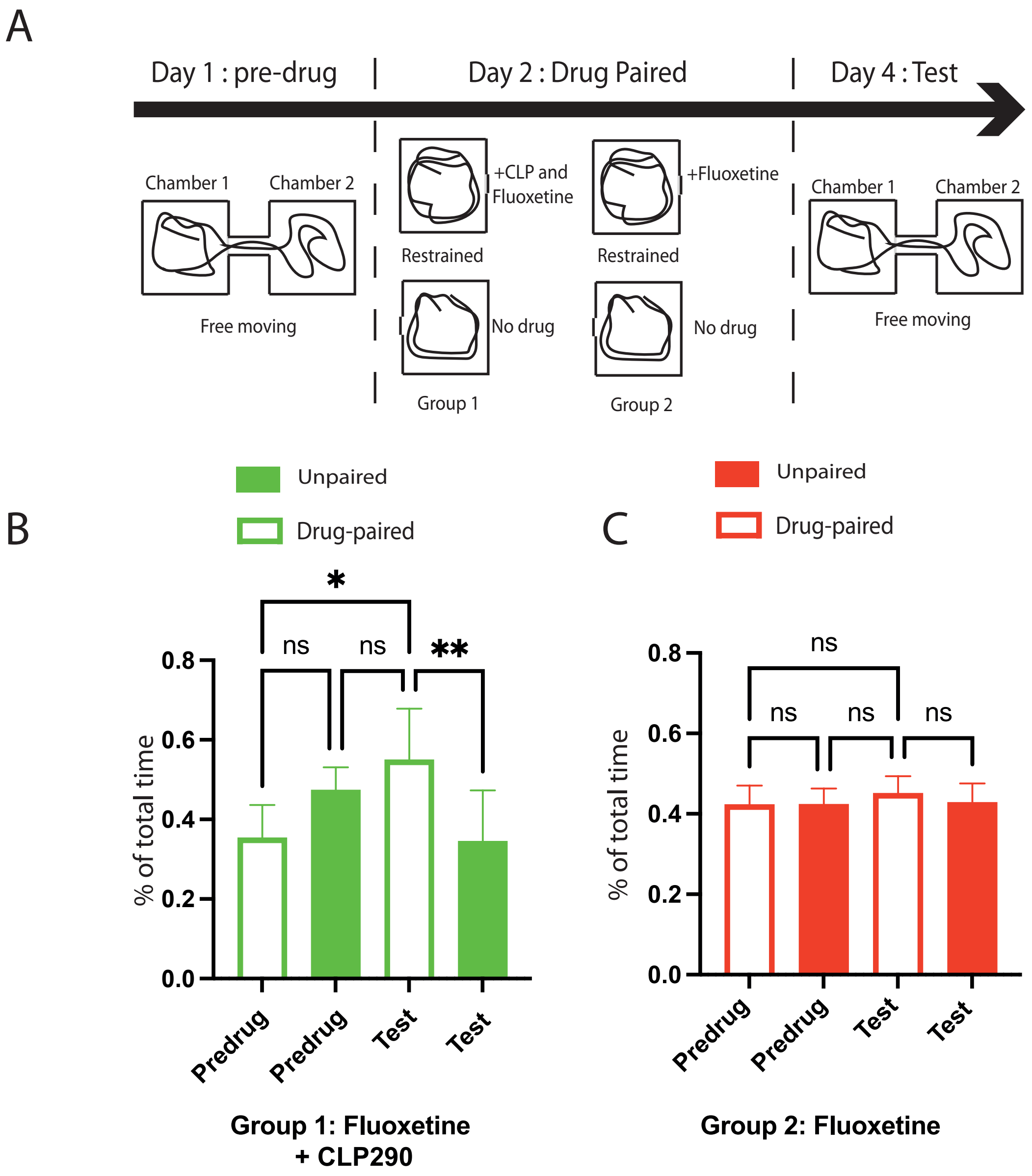

SupFig9. Aby et al 2022

Conditioned place preference (CPP) shows efficacy of combination fluoxetine and CLP290 in supraspinal pain components. A) Time course of CPP experiment. B) Treatment with fluoxetine and CLP290 significantly increased the time spent in the associated component. ($p<0.01$, Friedman test. Pre-drug (drug paired) vs test (drug-paired), $p<0.05$ and test (drug paired) vs test (unpaired), $p<0.01$, Dunn's multiple comparisons test). C) Treatment with fluoxetine alone did not modify the time spent in associated component. ($p=0.44$, Friedman Test).

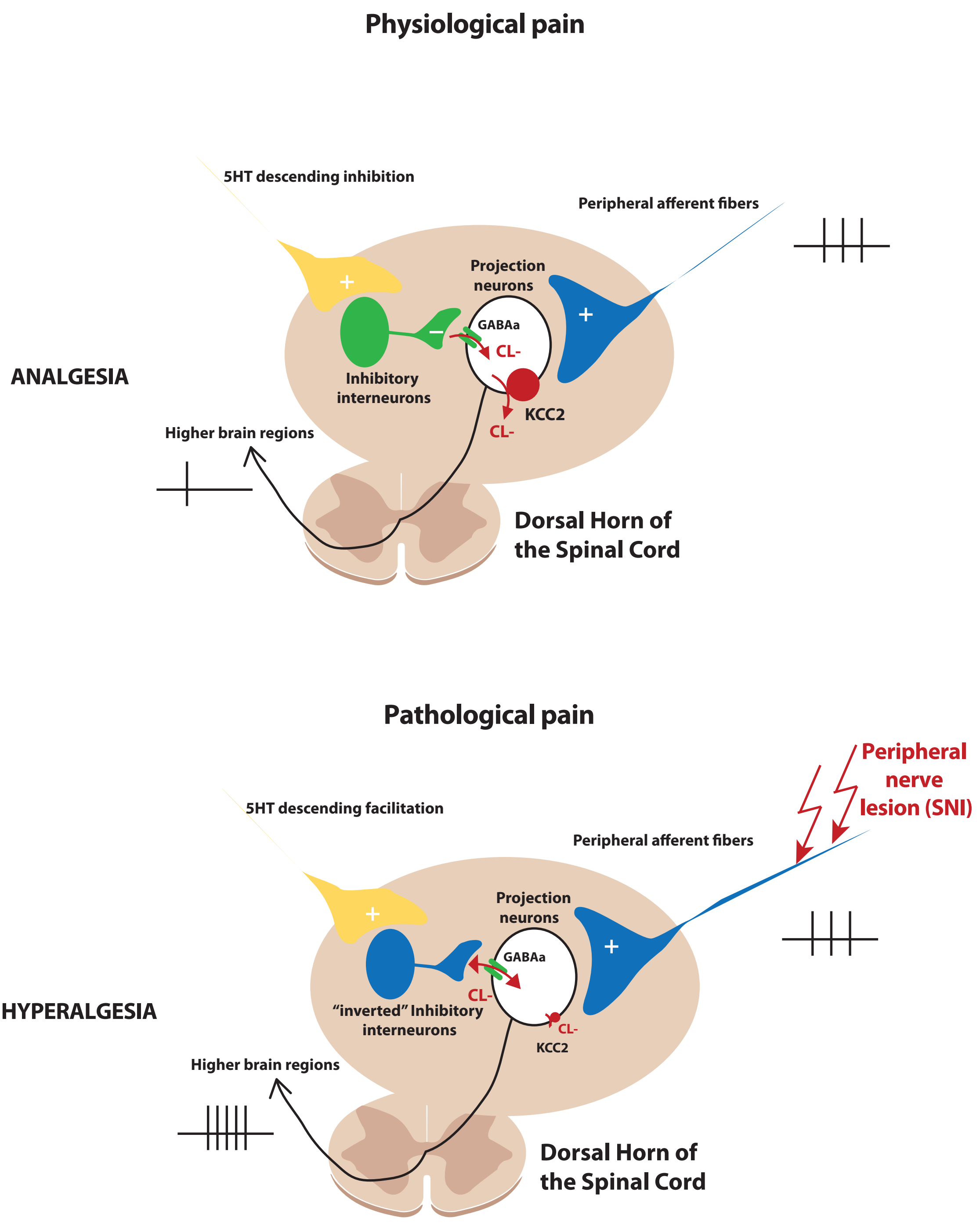

SupFig10. Aby et al. 2022

Global scheme of inverted 5HT descending pathway in naive mice and SNI model of mono-neuropathy.
